# Directed Evolution of the *Methanosarcina barkeri* Pyrrolysyl tRNA/aminoacyl tRNA Synthetase Pair for Rapid Evaluation of Sense Codon Reassignment Potential

**DOI:** 10.3390/ijms22020895

**Published:** 2021-01-18

**Authors:** David G. Schwark, Margaret A. Schmitt, John D. Fisk

**Affiliations:** Department of Chemistry, University of Colorado Denver, Campus Box 194, P.O. Box 173364, Denver, CO 80217-3364, USA; dschwark13@gmail.com (D.G.S.); margaret.schmitt@ucdenver.edu (M.A.S.)

**Keywords:** genetic code expansion, sense codon reassignment, pyrrolysyl tRNA, pyrrolysyl tRNA synthetase, synthetic biology, protein engineering, directed evolution

## Abstract

Genetic code expansion has largely focused on the reassignment of amber stop codons to insert single copies of non-canonical amino acids (ncAAs) into proteins. Increasing effort has been directed at employing the set of aminoacyl tRNA synthetase (aaRS) variants previously evolved for amber suppression to incorporate multiple copies of ncAAs in response to *sense* codons in *Escherichia coli.* Predicting which sense codons are most amenable to reassignment and which orthogonal translation machinery is best suited to each codon is challenging. This manuscript describes the directed evolution of a new, highly efficient variant of the *Methanosarcina barkeri* pyrrolysyl orthogonal tRNA/aaRS pair that activates and incorporates tyrosine. The evolved *M. barkeri* tRNA/aaRS pair reprograms the amber stop codon with 98.1 ± 3.6% efficiency in *E. coli* DH10B, rivaling the efficiency of the wild-type tyrosine-incorporating *Methanocaldococcus jannaschii* orthogonal pair. The new orthogonal pair is deployed for the rapid evaluation of sense codon reassignment potential using our previously developed fluorescence-based screen. Measurements of sense codon reassignment efficiencies with the evolved *M. barkeri* machinery are compared with related measurements employing the *M. jannaschii* orthogonal pair system. Importantly, we observe different patterns of sense codon reassignment efficiency for the *M. jannaschii* tyrosyl and *M. barkeri* pyrrolysyl systems, suggesting that particular codons will be better suited to reassignment by different orthogonal pairs. A broad evaluation of sense codon reassignment efficiencies to tyrosine with the *M. barkeri* system will highlight the most promising positions at which the *M. barkeri* orthogonal pair may infiltrate the *E. coli* genetic code.

## 1. Introduction

Genetic code expansion, biosynthetic incorporation of non-canonical amino acids (ncAAs) to expand the chemical properties of proteins, is a rapidly developing area of synthetic biology research [1,2]. Genetic code expansion has largely focused on the reassignment of stop codons to insert a single copy of a particular ncAA into a protein [3,4]. The most widely-employed strategy for the incorporation of ncAAs utilizes an evolved orthogonal aminoacyl tRNA synthetase (aaRS) and its cognate tRNA, which are expressed in the cells used to produce the modified protein of interest [5,6]. The orthogonal aaRS is evolved to recognize and attach an exogenously-supplied ncAA to the orthogonal tRNA. The orthogonal tRNA does not interact with the natural complement of aaRSs in the organism into which it is transplanted [7]. The orthogonal aaRS only recognizes its cognate tRNA and does not aminoacylate the organism’s endogenous tRNAs. The orthogonal pair exploits the phylogenetic distance between its endogenous organism and the organism in which the genetic code will be expanded to ensure specificity.

In the gene from which the protein of interest is expressed, an in-frame stop codon, typically the amber stop codon, is recognized by the anticodon of the orthogonal tRNA charged with the ncAA, and the ncAA is introduced. Nearly 200 different ncAAs have been incorporated biosynthetically by repeatedly evolving primarily two orthogonal tRNA/aaRS pairs [1]. The tyrosyl tRNA/aaRS pair from *Methanocaldococcus jannaschii* is orthogonal to the translation components of bacterial cells [6], and the pyrrolysyl tRNA/aaRS pair from *Methanosarcina* sp. (usually *Methanosarcina barkeri* or *Methanosarcina mazei*) is orthogonal to the translation components of bacterial, archaeal, and eukaryotic cells [8,9]. The use of stop codons as the targets of genetic code expansion has the advantage of allowing easy separation of truncated proteins produced when a missed incorporation results in termination of translation.

The ability to incorporate more than one type of ncAA in the same protein expands the space of potential genetic codes exponentially. Using combinations of already-evolved orthogonal aaRSs, thousands (~200^2) of 22 amino acid genetic codes are possible. However, the orthogonal ncAA-incorporating machinery is only one part of the equation. For every additional ncAA type incorporated into a given protein, an additional codon must be repurposed [2]. While one of the two remaining stop codons could be utilized for further genetic code expansion, competition with translation termination at each site substantially reduces the yield of full-length protein and renders multisite substitutions difficult. Systems using genomic editing and/or elongation factor engineering to mitigate the cellular reliance on the amber codon as a termination signal have expanded the utility of amber stop codon-mediated expanded genetic codes and made multisite incorporation of a single ncAA more readily achievable [10,11,12].

We and others have been interested in the long-term goal of employing the set of aaRS variants previously evolved to aminoacylate their cognate tRNA with a CUA anticodon (for decoding the UAG amber stop codon) to incorporate ncAAs in response to sense codons [13,14,15,16,17,18,19,20]. The genetic code is degenerate. In *E. coli*, 61 sense codons are read by 41 tRNAs and signal incorporation of the 20 canonical amino acids. For 18 of 20 amino acids, reassigning one sense codon to a ncAA leaves at least one other codon available to incorporate the canonical amino acid. Unlike stop codon repurposing, missed incorporations do not lead to truncated products. Although downstream separation of the protein mixture may be challenging (depending on the ncAA incorporated), a missed ncAA incorporation early in the sequence does not automatically preclude incorporation of ncAAs in response to targeted codons further along the mRNA. Sense codon reassignment should improve the ability to access proteins in which multiple copies of multiple ncAAs are biosynthetically incorporated. For many applications, multisite incorporation of ncAAs within a protein mixture could be desirable. For example, ncAA-dependent protein function could be selected from directed evolution libraries at incorporation efficiencies far less than 100%. The *M. jannaschii* and *M. barkeri* orthogonal tRNA/aaRS pairs are orthogonal to each other [21,22,23]. Several instances of dual ncAA incorporation utilizing both orthogonal pairs in response to stop and four-base codons have been reported [24,25,26]. Engineering systems in which evolved variants of the *M. jannaschii* and *M. barkeri* orthogonal tRNA/aaRS pairs each target different codons will facilitate biosynthesis of proteins simultaneously containing multiple copies of multiple ncAAs.

Unfortunately, predicting which sense codons are most amenable to reassignment and which orthogonal translation machinery is best suited to each codon is challenging. The fidelity of protein translation is governed by interactions between the tRNA and aaRS at the amino acid charging step as well as interactions between the aminoacylated tRNA, mRNA, and other translation components at the peptide bond formation step. The tRNA anticodon–mRNA codon recognition event drives the process. However, additional interactions outside of the anticodon contribute to the speed and specificity of acceptance of the tRNA in protein translation [27,28]. This suite of interactions and their relative quantitative importance in determining the thermodynamics and kinetics of protein translation have been only partially determined [7,29]. How this space of interactions may be infiltrated by orthogonal tRNA/aaRS pairs has not been broadly experimentally assessed. 

To quickly map the space of sense codon reassignment by the *M. jannaschii* Tyr pair, we repurposed the native function of the aaRS, charging Tyr to the cognate tRNA, and developed a simple fluorescence-based screen [17]. Tyrosine is an easy-to-evaluate surrogate for the nearly 200 non-canonical amino acids previously incorporated into proteins biosynthetically. Our screen exploits the absolute requirement of a tyrosine at position 66, flanked by Thr/Ser at position 65 and Gly at position 67, for autocatalytic formation of the green fluorescent protein (GFP) fluorophore. Incorporation of any other canonical amino acid in place of the active site tyrosine effectively abolishes fluorescence [30]. The screen quantitatively reports on the ability of the tyrosine-charging *M. jannaschii* aaRS to recognize *M. jannaschii* tRNAs with altered anticodons and the extent to which Tyr-aminoacylated *M. jannaschii* tRNAs successfully compete with endogenous *E. coli* tRNAs to decode each individual sense codon. We have evaluated reassignment of more than 30 *E. coli* codons and found that every sense codon evaluated could be partially reassigned simply by providing an orthogonal tRNA with an anticodon capable of Watson–Crick base pairing to the targeted codon [17,31,32,33]. This evaluation has identified promising sense codon targets for ncAA incorporation, revealed modification of the orthogonal tRNA by endogenous tRNA modification enzymes, facilitated improvement of sense codon reassignment via directed evolution, and provided a suite of related data with which to dissect the relative quantitative importance of the factors governing the ultimate selection of an aminoacylated tRNA.

Here we describe the directed evolution of a new variant of the orthogonal *M. barkeri* pyrrolysyl translation machinery for the rapid evaluation of sense codon reassignment using our previously reported fluorescence-based screen. Our fluorescence-based screen is useful not only for evaluating codon reassignment to tyrosine, but also to screen libraries for improved tyrosine incorporation. Using a combination of site-directed and random mutagenesis, we identify and characterize variants of the *M. barkeri* Pyl aaRS that charge tyrosine onto its cognate tRNA. The first phase of evolution utilized a library of aaRS variants with diversity elements targeted to several amino acid residues expected to play an important role in amino acid recognition. The aaRS library was co-transformed with the GFP reporter vector containing an amber UAG codon at position 66 and screened using fluorescence-activated cell sorting (FACS). After collection and amplification of cells displaying increased fluorescence, a tyrosine-charging aaRS variant, TyrGen1, was identified. The evolved aaRS and its cognate tRNA with a CUA anticodon reassigned the UAG stop codon with an efficiency of 8.4 ± 0.6% in *E. coli* DH10B. Mass spectrometry analysis of protein produced using this evolved orthogonal pair revealed predominantly incorporation of Tyr in response to the stop codon. A small amount of Phe incorporation was also observed in the mass spectrum.

Error-prone PCR was used to improve the function of the aaRS. FACS was again used to screen a large number of aaRS variants. Following rigorous characterization, we identified a variant of the *M. barkeri* Pyl aaRS, TyrGen2, that aminoacylates tyrosine onto its cognate tRNA with an efficiency rivaling the natural *M. jannaschii* enzyme. The second-generation orthogonal pair reassigns the amber stop codon with an efficiency of 98.1 ± 3.6% in *E. coli* DH10B. In addition to identifying an aaRS variant with improved catalytic efficiency, substrate specificity was also improved in TyrGen2. Phenylalanine incorporation was undetectable by mass spectrometry. 

We also report on the potential of sense codon reassignment by the *M. barkeri* pyrrolysyl tRNA/aaRS pair at four sense codons. As was the case with our evaluation of sense codon reassignment by the tyrosine-charging *M. jannaschii* aaRS and its tRNA, each sense codon we examined was partially reassigned by the tyrosine-charging *M. barkeri* Pyl aaRS and its tRNA simply by changing the anticodon of the tRNA to Watson–Crick base pair with the targeted codon. The *M. barkeri* tRNA was able to discriminate between the targeted, Watson–Crick base pairing codon and non-targeted codons with great fidelity.

Interestingly, we observe different patterns of sense codon reassignment efficiency between the tyrosine-incorporating versions of the *M. jannaschii* and *M. barkeri* orthogonal pairs, suggesting that a more complete evaluation of sense codon reassignments to tyrosine using the *M. barkeri* system reported here is warranted. Of particular interest is an opportunity to begin to isolate the contribution of aminoacylation efficiency to the fidelity of translation [34,35]. In the case of the *M. jannaschii* tyrosyl tRNA/aaRS pair, the tRNA anticodon is an important identity element for tRNA recognition by the aaRS [36,37]. Changes in the anticodon sequence required to target different sense codons differentially impact the efficiency of tRNA charging. In contrast, the *M. barkeri* aaRS does not rely upon the sequence of the anticodon as an identity element for tRNA recognition [38]. Comparing the efficiency of sense codon reassignments by the two orthogonal systems should begin to reveal the contribution of orthogonal tRNA aminoacylation efficiency to sense codon reassignment.

## 2. Results and Discussion

### 2.1. Directed Evolution of M. barkeri Pyl aaRS Variants that Amionoacylate Tyrosine

#### 2.1.1. Design and Preparation of a Library of *M. barkeri* Pyl aaRS Amino Acid-Binding Pocket Variants

The pyrrolysyl tRNA/aaRS pairs from both *M. barkeri* and *M. mazei* have been evolved for ncAA incorporation, and the two pairs share a great deal of similarity. The sequences of the pyrrolysyl tRNA from *M. barkeri* and *M. mazei* differ in sequence at only two bases: G3(Mb) vs. A3(Mm) and U48(Mb) vs. C48(Mm). Both tRNAs share a set of unusual features, including a long anticodon stem, abbreviated D and variable loops, and a missing hinge base [39]. Structures of the *M. mazei* tRNA show that the tRNA folds into a canonical L-shaped structure despite the atypical features [9,40,41]. Given their sequence similarity, the *M. barkeri* tRNA is expected to adopt a similar structure. Likewise, the protein sequences of the *M. barkeri* and *M mazei* aaRSs align, with two short gaps in the N-terminal domain. The *Methanosarcina* sp. pyrrolysyl aaRS is structurally related to type II synthetases and exists as a homodimer. Each monomer unit consists of three domains: an N-terminal domain that is involved in tRNA recognition, a small central linker domain, and a C-terminal domain, which catalyzes amino acid attachment to the tRNA [42]. Between the two organisms, the sequences of the C-terminal catalytic domain are 82% identical and 92% similar. Several crystal structures of the C-terminal domains of either the wild-type *M. mazei* Pyl aaRS or related evolved aaRSs which activate ncAAs have been reported [41,42,43,44,45,46]. 

The unusual features of both the *Methanosarcina* sp. pyrrolysyl tRNA and its cognate aaRS contribute to this pair’s broad organismal orthogonality [47]. The aaRS is different from other type II synthetases in that the tRNA-binding elements wrap around the tRNA and make very specific contacts with the tRNA body [48]. Structural studies on the *Methanosarcina* sp. pyrrolysyl aaRSs clearly show that no portion of the aaRS is in contact with the tRNA anticodon or anticodon loop [41,42,44,48]. The structural conclusions are bolstered by biochemical data that reveal minimal cross-reactivity with endogenous tRNAs across multiple species [41]. The tRNA body is itself different from all canonical *E. coli* tRNAs as a result of its long anticodon stem, abbreviated D and variable loops, and a missing hinge base. Even *E. coli* aaRSs that use the anticodon sequence as a primary identity element are expected to have very poor contacts with the *Methanosarcina* sp. pyrrolysyl tRNA body due to the unusual structural features. Additionally, regardless of the sequence of the engineered anticodon, the *Methanosarcina* sp. pyrrolysyl tRNA lacks at least some of the recognized identity elements for every *E. coli* aaRS and often includes *E. coli* aaRS anti-determinants [37].

Previous directed evolution experiments and the evolutionary history of the Pyl aaRS suggest that the development of enzymes able to recognize tyrosine should be possible. The two pyrrolysyl orthogonal tRNA/aaRS pairs have been evolved to accept several amino acids with side chains of similar shape to tyrosine including phenylalanine (Phe), O-methyltyrosine (OmeY) and 4-bromo/iodo-phenylalanine (pBr/pI-Phe) (Table 1) [45,49,50,51,52]. These aaRSs recognize the cognate tRNA^Pyl^ bearing a CUA anticodon for amber stop codon suppression. Additionally, the Pyl aaRS appears to be evolutionarily most closely related to phenylalanyl aaRSs [44]. 

To produce a variant of the *M. barkeri* Pyl aaRS able to charge tyrosine onto its cognate tRNA, a combination of structural and functional data was used to identify positions to vary as part of a large library of aaRS mutants. Given their high similarity, the *M. barkeri* and *M. mazei* pyrrolysyl aaRS structures are presumed to be functionally identical, and reports of directed evolution for ncAA incorporation using either aaRS were considered. A single fixed mutation (Y349F), previously shown to increase aminoacylation efficiency, was included in the library starting material [45]. (Full sequences of aaRSs are found in the Supplementary Material, S.5. Oligonucleotide sequences for gene construction and library diversity are found in Tables S1–S3).

The library targeted positions surrounding the amino acid-binding pocket of the C-terminal catalytic domain. Six amino acid residues that were found to vary across the suite of *Methanosarcina* sp. Pyl aaRS variants that recognize aromatic amino acids were selected (Table 1). The side chains of these residues appear to line the amino acid-binding pocket in crystal structures of the C-terminal domain of the *M. mazei* aaRS bound to pyrrolysine [42,44]. The degenerate NNK codon was used in oligonucleotide primers to vary Ala 267, Tyr 271, Leu 274, Asn 311, Cys 313 and Val 367 (*M. barkeri* numbering). The NNK codon encodes all 20 amino acids and 1 stop codon. The designed library had a theoretical diversity of 1.1 × 10^9^ at the DNA level and 6.4 × 10^7^ at the protein level.

The library was prepared using Kunkel mutagenesis with three mutagenic primers [53,54]. The library template included restriction sites within the aaRS that were removed by the mutagenic primers. The initially prepared and transformed library included 1.01 × 10^9^ unique transformants, covering the theoretical diversity of the library. Following library creation, an additional DNA purification step was undertaken to enable restriction digestion of unmutated and partially mutated template, reducing the background in the library. The resultant DNA was transformed into cells harboring a GFP reporter vector with an amber stop codon at position 66. Restriction analysis and sequencing of randomly picked clones after digestion and transformation revealed minimal carry-through of template DNA: 19/23 clones were unique full library members. The entire directed evolution workflow is depicted visually in Supplementary Figure S1.

#### 2.1.2. High-Throughput Screening of the Targeted aaRS Library and Amplification of Apparent Tyrosine-Charging aaRS Variants

After induction with IPTG, 1.2 × 10^8^ cells were sorted using fluorescence-activated cell sorting (FACS). FACS-based screens are increasingly being used over selection-based approaches to evolve orthogonal tRNA/aaRS pairs [17,56,57,58,59,60,61,62]. Unsurprisingly, most of the cells screened in the first round of FACS did not aminoacylate tyrosine onto the *M. barkeri* tRNA and did not produce full-length, fluorescent GFP. The distribution of fluorescence included a long tail with a population of cells with detectable fluorescence greater than that of the non-fluorescent control population (Supplementary Figure S2). Cells displaying fluorescence within the top 1% of fluorescence values (approximately 0.03%) were collected, amplified, and resorted. 

The cell population screened in the second round of FACS was enriched in cells with detectable fluorescence. Cells with intermediate fluorescence (1.3 × 10^4^ cells, approximately 0.07% of the population) were collected separately from those with very high fluorescence. During the second round of FACS, a population of cells with fluorescence similar to that of wild-type GFP, profiled as a control, was observed (Supplementary Figure S2). The two fluorescent populations were collected separately in an effort to segregate cells with possible fluorophore revertants from cells with functional aaRSs. In previous high-throughput, GFP-based gain of function screens, false positives arising from either GFP fluorophore revertants (UAG stop codon to either UAU or UAC) or mutations that led to an increase in the expression of either the GFP reporter or aaRS have been observed [17].

A portion of the cells with intermediate fluorescence were plated prior to amplification for analysis of individual clones. Several clones appeared to aminoacylate and incorporate Tyr in response to the amber stop codon at position 66 in the reporter. Of 24 evaluated clones, 23 appeared to reassign the UAG codon to Tyr at a level above background (~0.15%) and below wild-type GFP. The remaining clone had very high apparent reassignment efficiency, suggestive of a fluorophore revertant. This clone was not analyzed further.

#### 2.1.3. Characterization of *M. barkeri* Pyl Aminoacyl tRNA Synthetase Variants Confirms Incorporation of Tyrosine in Response to the Amber Stop Codon

To confirm aaRS activity, additional characterization of nine individual clones with an apparent reassignment efficiency greater than 7.0% was undertaken. The orthogonal translation machinery vector was isolated and retransformed with non-sorted GFP reporter DNA for analysis of biological replicates in the fluorescence-based screen. aaRS activity was further verified by PCR amplifying the aaRS gene out of the backbone isolated from the FACS-collected cells, cloning the aaRS into vector DNA that had not been cycled through any screening, and evaluating the performance in the fluorescence-based screen. After stringent verification of enzyme activity, one unique DNA sequence, TyrGen1, displayed reproducible reassignment activity. 

The TyrGen1 *M. barkeri* aaRS variant reassigns the amber stop codon within the GFP reporter to tyrosine with an efficiency of 8.4 ± 0.6% in DH10B cells. Four of six amino acid positions allowed to vary as part of the library were mutated relative to the wild-type aaRS in TyrGen1 (Table 1). Across the sequences of the aaRS variants used to guide library design, the identity of the amino acid at two positions, 267 and 274, typically did not change from the wild-type sequence as the enzyme was evolved. Both positions were mutated in TyrGen1. Lys 267 has not been observed in the aaRSs specific for other amino acids [1]. Two positions, Tyr 271 and Val 367, did not change between the wild-type sequence and TyrGen1. Position 313 was different in each of the five sequences used to guide our library design and in TyrGen1. The remaining position, 311, tended to be a small amino acid side chain across the aaRS sequences compared.

### 2.2. System Modifications Improve the Amber Reassignment Efficiency of the Evolved aaRS

Decoding an amber stop codon is expected to be one of the more efficient functions of an orthogonal tRNA because the orthogonal tRNA’s competition is against protein termination machinery as opposed to endogenous tRNAs. In many organisms, stop codons, typically the amber codon, are partially decoded as sense codons by suppressor tRNAs [63]. In fact, pyrrolysine is incorporated into proteins in *Methanosarcina* sp. by an amber-suppressing tRNA [38,64]. 

The *M. jannaschii* tyrosyl tRNA/aaRS pair introduces Tyr in response to an amber stop codon with efficiencies between 80% and 90% in the fluorescence-based screen, depending on the *E. coli* strain used [17,33]. The high efficiency of amber suppression leads to a very large dynamic range over which to evaluate the reassignment of sense codons by the *M. jannaschii* pair. The dynamic range afforded by the *M. barkeri* aaRS evolved for Tyr incorporation (efficiency 8.4 ± 0.6%) is much smaller. Improving the function of the orthogonal pair to expand the dynamic range is necessary for quantifying the more infrequent instances of reassignment by the orthogonal tRNA. 

Unlike the *M. jannaschii* tRNA/aaRS pair, which naturally aminoacylates and incorporates Tyr, the evolved tyrosine-incorporating *M. barkeri* tRNA/aaRS pair is not expected to be particularly efficient. Engineered aaRSs are typically not as efficient as natural aaRSs. The efficiencies of ncAA incorporation in response to stop codons by engineered aaRSs average about 25% per position [65]. The observed incorporation efficiencies are the end result of multiple factors that affect translation. In the case of some ncAAs, transport of the amino acid into the cell may further limit incorporation efficiency [66]. At 8.4 ± 0.6%, TyrGen1 displays similar efficiency to many natural amber suppressors and to aaRSs evolved for ncAA incorporation in response to amber stop codons [63,67].

For a given orthogonal tRNA/aaRS pair, reassignment efficiencies may be increased via either systemic or functional modifications. Systemic modifications involve alterations of molecular component concentrations (e.g., tRNA, aaRS, amino acid substrate) or environmental conditions (e.g., media composition). Functional modifications focus on improving the molecular function of each of the components, such as the catalytic efficiency of the aaRS, the molecular recognition between the tRNA and aaRS, and/or the efficiency of interaction between the aminoacylated tRNA and the organism’s translation machinery. Efficiency increases as a result of systemic and functional mechanisms should be additive.

**Figure 1:**
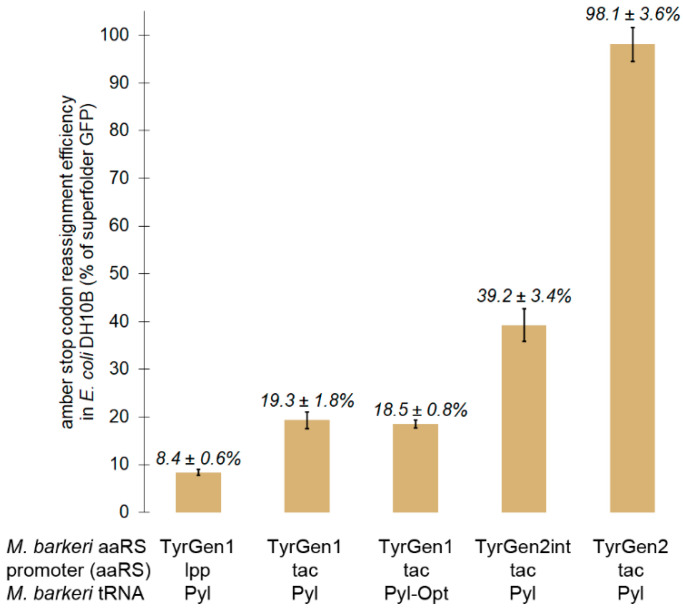
Progression of the efficiency of amber stop codon reassignment by evolved tyrosine-incorporating *M. barkeri* Pyl aaRS variants measured in *E. coli* DH10B. Efficiencies are presented as a percentage of the fluorescence of cells producing GFP with a Tyr codon specifying position 66. The table below the x axis reports the iteration of aaRS, the promoter driving aaRS expression, and the tRNA that give rise to the reported reassignment efficiency. For each evaluation, a GFP reporter vector with the UAG codon specifying the tyrosine residue required for GFP fluorophore formation is combined with a vector expressing the orthogonal aminoacyl tRNA synthetase and its cognate tRNA with a CUA anticodon. Efficiencies are calculated relative to a “100% fluorescence control”, a GFP reporter with a tyrosine codon specifying the fluorophore, and a non-fluorescent “0% fluorescence control”, a GFP reporter with a non-tyrosine codon specifying the fluorophore.

We evaluated the extent to which increasing the strength of the promoter driving transcription of the aaRS mRNA improved reassignment efficiency. Expression of the aaRS in the library vector was driven by the *lpp* promoter. The stronger *tac* promoter drives expression of the aaRS in the *M. jannaschii* tRNA/aaRS pair vector used to evaluate sense codon reassignment [17,33]. Replacing the *lpp* promoter with the *tac* promoter more than doubled the incorporation efficiency of tyrosine in response to amber stop codons by the *M. barkeri* tRNA/TyrGen1 aaRS pair to 19.3 ± 1.8% (Figure 1). 

Fan and co-workers examined the ways in which improved interactions between the orthogonal *M. barkeri* tRNA and *E. coli* EF-Tu translated to improved amber codon reassignment [68]. A variant of the *M. barkeri* tRNA, named tRNA^Pyl-Opt^, was found to improve incorporation of ncAAs in response to amber stop codons [68]. The most significant improvements in efficiency came as a result of mutations in the acceptor and T stems, portions of the tRNA that interact with EF-Tu. Expressed in concert with TyrGen1 and under the conditions of our screen, tRNA^Pyl-Opt^ did not increase the incorporation of Tyr in response to the UAG codon. The measured efficiency with tRNA^Pyl-Opt^ was 18.5 ± 0.8%, (Figure 1). 

EF-Tu–tRNA interactions are balanced such that both the amino acid and tRNA body contribute significantly to achieve uniform tRNA binding [69,70]. An apparent instance of overstabilization of EF-Tu–tRNA interactions interfering with ncAA incorporation has been described [71]. Aromatic amino acids have been shown to contribute strongly to EF-Tu affinity. In the case of our evolved tyrosine-activating system, improvement of the tRNA body interaction may not positively affect the overall efficiency of amino acid incorporation [72].

### 2.3. Random Mutagenesis Further Improves the Efficiency of Tyrosine Incorporation by the Evolved aaRS

The initially identified *M. barkeri* Tyr-activating aaRS variant, TyrGen1, represents a starting point for further improvement by directed evolution. Unlike the first round of aaRS evolution which was focused on amino acid residues that contribute to substrate specificity, the amino acid positions at which mutation might lead to increased efficiency of the aaRS are not obvious. In addition to improved substrate recognition, increased enzyme activity could arise from improvements to overall enzyme stability, catalytic activity, and/or expression. Error-prone PCR (EP-PCR) facilitates the introduction of random mutations across the entire parent aaRS sequence. Several early protocols for aaRS evolution employed rounds of error-prone PCR, [73,74], but the majority of aaRS directed evolution procedures have relied on cassette mutagenesis and not included rounds of error-prone PCR or gene shuffling. Recently, increased instances of the use of random mutagenesis for enzyme maturation have been reported [58,60].

The parent aaRS, TyrGen1, was amplified using unequal concentrations of nucleotide building blocks and reaction conditions designed to lower the fidelity of the thermostable Taq polymerase. The resulting PCR products were cloned into the backbone vector driven by the *tac* promoter in place of the KpnI-containing aaRS variant for concomitant digestion of unreacted starting material. Transformation of the aaRS EP-PCR library 1 into cells harboring the UAG reporter yielded 1.7 × 10^7^ unique transformants. 12/12 clones randomly selected for analysis were library members as judged by the stability of the aaRS DNA upon restriction digestion. Sequencing of the aaRSs from these clones revealed an average mutation rate of 2.2 mutations per 1000 nucleotides. Following induction of the cells with IPTG, 1.0 × 10^7^ cells were screened via FACS. Cells with fluorescence representing the top 1% (~0.4% of the population) were collected and amplified. Experimental details for the first round of EP-PCR are found in Table S4. A small portion of these cells were plated for isolated clone analysis. Of 48 individual clones analyzed, the majority (>30) appeared to decode UAG as tyrosine with efficiency similar to that of TyrGen1 (~20 ± 5%). Eight clones demonstrated appreciably improved reassignment efficiency (≥30%). One clone, TyrGen2int, was characterized further and reassigned the amber stop codon to Tyr with 39.2 ± 3.4% efficiency (Figure 1). Eight clones were less efficient (<15% reassignment efficiency). 

DNA from the pool of cells amplified after FACS analysis of the first EP-PCR library was isolated and used as the template for a second round of EP-PCR. 4.0 × 10^6^ unique transformants resulted. Again, approximately 1.0 × 10^7^ cells were screened via FACS, and the top 1% were collected. Experimental details for the second round of EP-PCR are found in Table S5. A portion of the collected cells were plated for analysis of individual clones. Nine of 24 clones analyzed had an apparent efficiency similar to that of the TyrGen1 aaRS (~20 ± 5%). Eleven clones reassigned the UAG codon to Tyr with efficiency significantly greater than that of TyrGen1. Five clones were considerably less efficient. As with the characterization of TyrGen1, aaRS variants identified as improved after FACS were rigorously evaluated by recloning to rule out false positives.

The most efficient aaRS identified after EP-PCR round 2, TyrGen2, enables its cognate tRNA to reassign the UAG codon with 98.1 ± 3.6% efficiency in *E. coli* DH10B (Figure 1). This efficiency rivals that of the naturally-evolved tyrosine-incorporating *M. jannaschii* tRNA/aaRS pair and is considerably more efficient than typical natural stop codon-suppressing pairs. The amino acids identified in the first aaRS variant that altered the substrate specificity of the enzyme to accept Tyr did not change in the second generation aaRS. Five additional amino acid mutations increased the efficiency of the enzyme nearly 5-fold: T20S, S121C, K125R, M265T, and E409V. Of these five positions, only residue 265 is near the amino acid substrate-binding pocket [42]. Three mutations—T20S, S121C, and K125R—are in the N-terminal domain of the protein. The side chain of the amino acid at position 20 does have contact with the tRNA body, and the conservative Thr to Ser mutation maintains the hydroxyl group which appears to be involved in that interaction [48]. Positions 121 and 125 are in a region of the aaRS between the N- and C-terminal domains for which no structural information is available. The region of the enzyme containing amino acid 409 may be involved in properly orienting the C-terminal tRNA-binding domain [41,42]. Position 409 does not appear to be involved in the tRNA-binding interface. Without a more detailed structure-function analysis, firm conclusions about the mechanisms driving the effect of these mutations on aaRS function are not possible.

### 2.4. Mass Spectrometry Confirms Incorporation of Tyrosine by the Evolved Aminoacyl tRNA Synthetases

The fidelity of the genetic code is protected by a highly evolved, complex system of amino acid discrimination and editing mechanisms. Across all forms of life, aaRSs discriminate based on amino acid size and polarity. Amino acids larger than the target substrate are excluded from the aaRS’s amino acid-binding pocket. Amino acids that are smaller than the correct substrate may fit into the pocket, but aminoacylation is typically disfavored for energetic or kinetic reasons. In the event that an incorrect amino acid is charged to the tRNA, many aaRSs have editing domains that evaluate the identity of the amino acid prior to release of the tRNA from the tRNA/aaRS/amino acid complex. Editing of incorrect amino acids from tRNAs often proceeds through a double-sieve mechanism [75]. The editing domains tend to have a binding pocket that is smaller than the correct amino acid. tRNAs incorrectly acylated with amino acids smaller than the cognate amino acid can enter the editing domain and are de-acylated. 

aaRSs engineered to recognize alternative amino acid substrates do not have millions of years of evolutionary history over which the fidelity of aminoacylation has been finely tuned. In contrast to natural enzymes, a general property of evolved aaRSs is promiscuity [76]. Many aaRSs evolved for ncAA incorporation are capable of recognizing and acylating multiple amino acids (canonical or non-canonical) with side chain shape similar to that of their target. The mutations that enabled the *M. barkeri* aaRS variant to accept Phe informed the design of the library from which the aaRS with activity for Tyr was identified. The Pyl aaRS itself is evolutionarily related to the family of Phe aaRSs. Recognition of other canonical amino acids, particularly Phe, by the aaRS evolved for tyrosine is a possibility.

The fluorescence-based screen reports only on incorporation of tyrosine, as incorporation of other canonical amino acids at position 66 in GFP give rise to proteins with either greatly reduced and shifted or no fluorescence. Electrospray ionization mass spectrometry (ESI-MS) was used to evaluate the identity of the amino acids incorporated in response to a single UAG codon in the Z domain of protein A [77]. 

The Z domain is a small, soluble, 8.3 kDa, three-helix protein that has been previously used to evaluate codon reassignment [18,78]. Position 5 of the Z domain has been shown to be a permissive site for amino acid substitution. The identity of the amino acid(s) incorporated in response to a UAG codon at position 5 demonstrate the substrate specificity of the *M. barkeri* aaRS variants reported here. The Z domain variants were co-expressed with the *M. barkeri* machinery to reassign the UAG codon to tyrosine in *E. coli* DH10B. After hexahistidine tag-based purification, intact proteins were analyzed using ESI-MS followed by deconvolution of the mass spectra using the Maximum Entropy algorithm (MassHunter Software, Agilent Technologies). A Z domain variant with a UAC codon at position 5 provides the mass profile for the presence of Tyr at position 5. 

**Figure 2:**
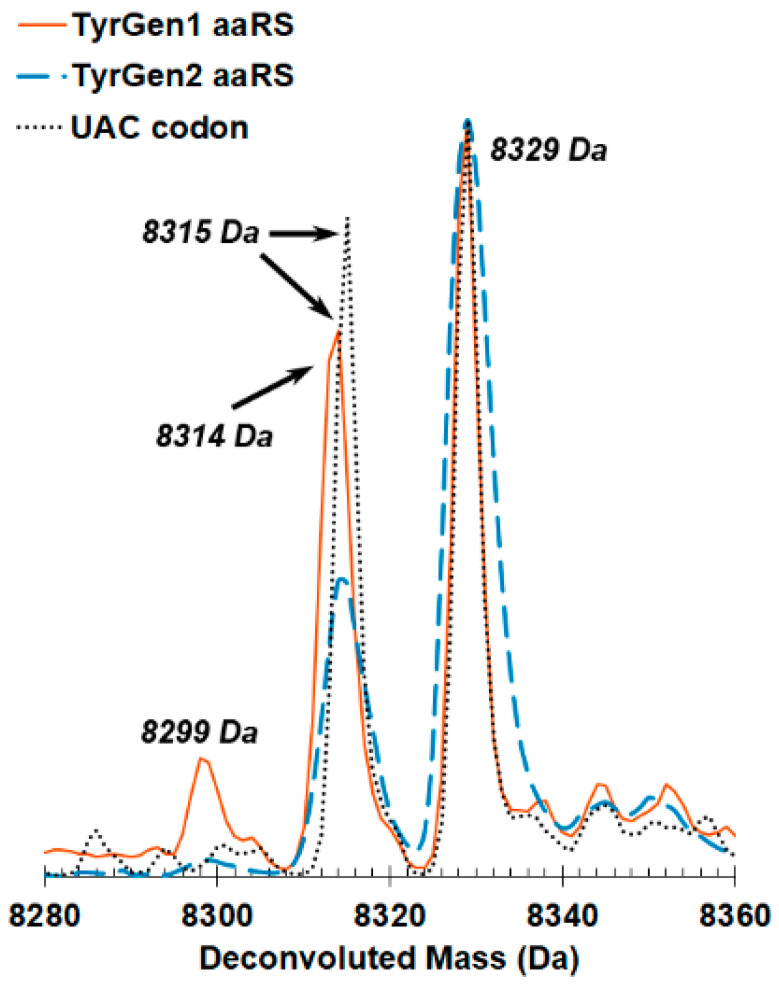
ESI-MS of purified Z domain proteins for identification of amino acids incorporated by evolved *M. barkeri* Pyl aminoacyl tRNA synthetases. Protein was translated via decoding a UAG amber stop codon (TyrGen1 aaRS: orange, solid line. TyrGen2 aaRS, blue, dashed line) or decoding a UAC Tyr codon (grey, dotted line). The calculated mass of the Z domain protein with tyrosine at position 5 is 8315 Da. This peak is observed in all three samples analyzed. A second +14 Da peak is also observed and likely corresponds to methylation of the Z domain. A second parent peak at 8299 Da in the mass spectrum of the full-length Z domain produced with the TyrGen1 aaRS suggests a small amount of phenylalanine incorporation. The observation of a broadened peak at 8314 Da and 8315 Da in this spectrum only is indicative of methylated Phe-incorporated Z domain (8314 Da) and non-methylated Tyr-incorporated Z domain (8315 Da). The second parent peak at 8299 Da is absent from the mass spectrum of the full-length Z domain produced after expression of the TyrGen2 aaRS, suggesting improved substrate specificity for Tyr.

The calculated mass of the Z domain protein with Y5 is 8315 Da, which is observed as the parent peak for all three protein samples (Figure 2). The second +14 Da peak observed for all parent peaks is likely due to a methylation of the Z domain [79].

A second parent peak at 8299 Da was also observed in the full-length protein translated in cells expressing TyrGen1. The -16 Da peak suggests a small percentage of incorporation of phenylalanine in response to the UAG codon. The mass of methylated Z domain with Phe at position 5 is 8313 Da. The broad peak at approximately 8314 Da likely represents both methylated F5 Z domain and unmethylated Y5 Z domain. Although mass spectrometry is not quantitative, the relative size of the parent peaks for the two proteins suggest that Tyr is a better substrate for TyrGen1 than Phe. 

Importantly, the peak at 8299 Da indicative of phenylalanine incorporation was absent from the mass spectrum of the protein produced in the presence of TyrGen2, suggesting that the mutations identified by error-prone PCR improved both the function and specificity of the aaRS for Tyr.

### 2.5. Application of Evolved Tyrosine-Charging aaRS Variants for Rapid Evaluation of the Efficiency of Sense Codon Reassignment

#### 2.5.1. The Tyrosine-Incorporating *M. barkeri* Pyl tRNA/aaRS Pairs Reassign Sense Codons Simply by Altering the Anticodon of the tRNA to Watson–Crick Base Pair with the Targeted Codon

We selected four sense codons, Lys AAG, Asn AAU, Glu GAG, and Arg AGG for an initial evaluation of sense codon reassignment by the *M. barkeri* tRNA/aaRS pair. Three codons—Lys AAG, Asn AAU, and Glu GAG—are read by endogenous *E. coli* tRNAs via wobble interactions. Wobble codons are an attractive target for genetic code expansion because differences in anticodon–codon binding energies between the introduced orthogonal tRNA, capable of Watson–Crick base pairing to the targeted codon, and the organism’s complement of tRNAs are expected to bias tRNA selection and amino acid incorporation [14]. Arg AGG is a rarely used codon throughout the *E. coli* genome and its tRNA, with a CCU anticodon, is of low abundance. Rarely used codons are an attractive target for breaking the degeneracy of the genetic code because the number of endogenous tRNAs against which the orthogonal tRNA must compete to decode the targeted codon tends to be low. Additionally, the Arg AGG codon has already been explored for ncAA incorporation using derivatives of the *Methanosarcina* sp. Pyl orthogonal pair [18,19,80]. 

In order to facilitate direct comparison to our full suite of sense codon reassignment efficiencies by the *M. jannaschii* orthogonal pair, the *E. coli* strain was switched from DH10B to SB3930 [17,32,33]. In *E. coli* SB3930, the efficiency of UAG reassignment by the TyrGen2 tRNA/aaRS pair decreased from 98.1 ± 3.6% to 65.2 ± 1.7%. Differences in incorporation efficiency between strains of *E. coli* despite using identical vectors and system conditions (e.g., media, antibiotics) have been observed anecdotally. Although some cell strain differences (e.g., knock-out of competing tRNA gene, removal of amber stop codons) are made with the intention of altering incorporation efficiency, it is not obvious why the *M. barkeri* system would be less efficient in SB3930 (a histidine auxotroph) compared to DH10B. All reported reassignment efficiencies represent data gathered for at least 6 biological replicates (Table S6).

The efficiency of reassignment at each codon was quantified using both TyrGen1 and TyrGen2. Each orthogonal translation machinery vector includes the *M. barkeri* aaRS (TyrGen1 or TyrGen2) and the tRNA expressed from identical promoters under identical conditions. The only difference between the suite of vectors for a given aaRS variant is the sequence of the anticodon in the tRNA. As a result, expression levels of the orthogonal tRNA and aaRS are expected to be nearly identical across compared systems.

**Figure 3:**
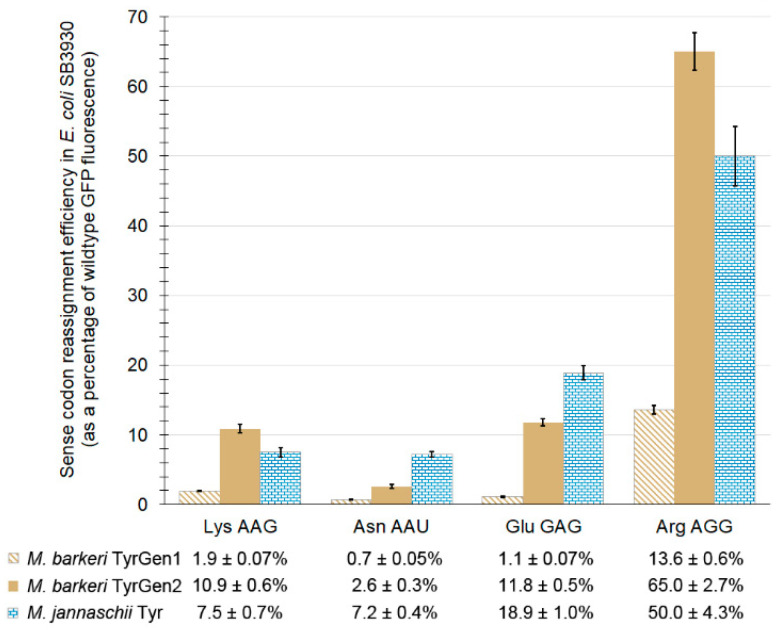
Reassignment efficiency of four sense codons in *E. coli* SB3930 by three orthogonal tRNA/aaRS pairs: *M. barkeri* TyrGen1 (gold, partly shaded bars); *M. barkeri* TyrGen2 (gold, fully shaded bars); *M. jannaschii* Tyr (blue, partly shaded bars, previously reported [17,32,33]). For each evaluation, a GFP reporter vector with the targeted codon specifying the tyrosine residue required for GFP fluorophore formation is combined with a vector expressing the orthogonal aminoacyl tRNA synthetase specified and its cognate tRNA with an anticodon capable of Watson–Crick base pairing to the targeted codon. Efficiencies are calculated relative to a “100% fluorescence control”, a GFP reporter with a tyrosine codon specifying the fluorophore, and a non-fluorescent “0% fluorescence control”, a GFP reporter with a non-tyrosine codon specifying the fluorophore.

**Figure 4:**
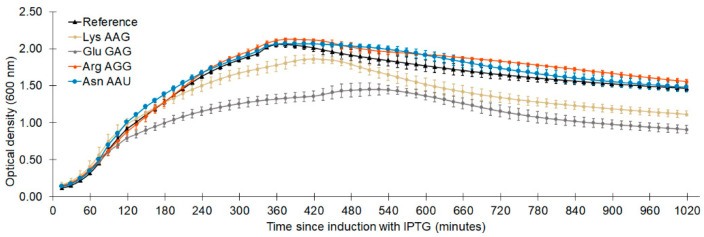
Optical density versus time since induction with IPTG profiles for sense codon reassigning systems expressing the TyrGen2 orthogonal pair. The reference system combined a non-fluorescent GFP reporter vector with an orthogonal translation machinery vector expressing an inactive version of the Pyl aaRS and the tRNA with a CUA anticodon.

Each codon evaluated could be reassigned to some extent, as was found in our parallel evaluation using the *M. jannaschii* pair (Figure 3). Efficiencies of sense codon reassignment range from 0.7 ± 0.05% to 13.6 ± 0.6% for the TyrGen1 aaRS and 2.6 ± 0.3% to 65.0 ± 2.7% for the TyrGen2 aaRS. The reassignment of each sense codon using the TyrGen2 aaRS improved between approximately 4- to 11-fold relative to that measured using the TyrGen1 aaRS, similar to the 5-fold improvement observed for reassignment of the amber stop codon between the two aaRSs. Sense codon reassignment has a minor impact on cellular fitness, as we and others have observed [17,31,32,81]. For the four sense codons examined here, proteome-wide partial reassignment using the TyrGen2 pair decreased cellular fitnesses to between 86 and 96% that of a similarly burdened but non-reassigning reference system, as determined by the ratio of instantaneous doubling times (Table 2). Carrying capacity and growth profiles for these sense codon reassigning systems showed idiosyncratic effects (Figure 4). Instantaneous doubling times and relative system fitnesses for systems expressing the TyrGen2 pair were calculated as previously described [31].

#### 2.5.2. Divergent Efficiencies of Sense Codon Reassignment by the *M. barkeri* and *M. jannaschii* Orthogonal Pairs Suggest Broad Evaluation of Sense Codon Reassignment by the *M. barkeri* tRNA/aaRS Pair will be Informative

Across the five codons evaluated, three (Asn AAU, Glu GAG, amber UAG) are reassigned more efficiently by the *M. jannaschii* orthogonal pair. Two, Lys AAG and Arg AGG, are reassigned more efficiently by the *M. barkeri* TyrGen2 pair. The observation of divergent patterns of codon reassignment efficiency is likely indicative of differences in the activity of the aaRSs with respect to aminoacylation of the cognate tRNA. The tRNA anticodon is an important identity element for tRNA recognition by the aaRS in the *M. jannaschii* tyrosyl tRNA/aaRS pair [36]. In contrast, the *M. barkeri* aaRS does not rely upon the sequence of the anticodon as an identity element for tRNA recognition [38]. The impact of single nucleotide changes in the *M. jannaschii* tRNA anticodon on the efficiency of aminoacylation by the *M. jannaschii* Tyr aaRS have been quantified and shown to be mostly additive [36]. A more complete evaluation of sense codon reassignments to Tyr by the *M. barkeri* orthogonal pair is expected to facilitate dissection of the contribution of tRNA aminoacylation efficiency to sense codon reassignment and the fidelity of the genetic code.

#### 2.5.3. The Tyrosine-Incorporating *M. barkeri* Pyl tRNA/aaRS Pairs Discriminate between Targeted and Non-Targeted Codons

The fluorescence-based screen provides a facile method for evaluating specific off-target decoding events by the orthogonal tRNA, simply by co-transforming the orthogonal translation machinery with a GFP reporter with a different codon specifying the fluorophore Tyr. Of particular import is the ability to quantify the extent to which each sense codon targeting tRNA is able to decode non-targeted, near cognate codons, typically codons which differ from the targeted codon at only the third position. Though infrequent for many tRNAs, decoding non-targeted codons is relevant for understanding the structure and function of natural translation systems and engineering the infiltration of the genetic code for the incorporation of ncAAs. 

In our previous analysis of codon discrimination by the suite of *M. jannaschii* tRNAs with altered anticodons, we detected some off-target codon readings. Occasionally, even energetically unfavorable A34/C3 and C34/A3 decoding was observed [17,31,32]. We evaluated the anticodon-altered *M. barkeri* tRNAs for reassignment of the alternative Lys AAA, Asn AAC, Glu GAA, and Arg AGA codons and found that they discriminated between their targeted and non-targeted codons with ratios greater than 95:5 (Table 3). Three of the four measurements were below the detection limit of the in cell assay (0.15%).

## 3. Materials and Methods 

The Supplementary Materials file includes general reagents and materials, as well as detailed experimental protocols for gene construction (primer sequences in Tables S1 and S2) [82], site-directed (primer sequences in Table S3) [53,54] and error-prone PCR mutagenesis (conditions summarized in Tables S4 and S5) [83,84], the fluorescence-based screen, and protein expression and purification for mass spectrometry. This file also includes a graphical representation of the directed evolution workflow (Figure S1) and traces from the FACS screening of the TyrGen1 aaRS (Figure S2). Finally, further experimental information, including cell strain information, numbers of biological replicates analyzed for each codon reassignment system (Table S6), and preparation of electrocompetent cells [85] are provided.

## 4. Conclusions

We used a combination of site-directed and random mutagenesis to evolve a variant of the orthogonal *M. barkeri* pyrrolysyl aminoacyl tRNA synthetase capable of charging its cognate tRNA with tyrosine with an efficiency rivaling that of natural aaRSs. After screening a targeted library of amino acid-binding pocket variants, the moderately efficient and specific aaRS was improved in both efficiency and specificity through two rounds of random mutagenesis. This success, like other recent reports, suggests that the genetic code expansion community is collectively coming to the realization that evolution of orthogonal translation machinery is enhanced when the process parallels that of the innate immune system for the generation of antibodies: an initial selection for activity, here via site-directed mutagenesis, followed by a process of maturation, via error-prone PCR and/or gene shuffling.

A preliminary application of the evolved *M. barkeri* pairs in our previously reported fluorescence-based screen for sense codon reassignment suggests that, as we observed with the parallel evaluation using the *M. jannaschii* pair, the degeneracy of the genetic code is breakable at many sense codons simply by providing an orthogonal tRNA with an anticodon capable of Watson–Crick base pairing to the targeted codon. The points within the genetic code which are most susceptible to infiltration and the orthogonal pair best suited to reassignment of each codon are not obvious *a priori*. Trends in reassignment efficiencies were not consistent between the *M. jannaschii* and *M. barkeri* orthogonal pairs and suggest that a more complete evaluation of sense codon reassignment by the newly-evolved *M. barkeri* tRNA/aaRS pair is warranted. Although the primary difference between the two pairs is expected to be the effect of changes to the anticodon on the enzymatic efficiency of the aaRS, additional interactions between the two unique orthogonal tRNAs and the rest of the endogenous translation machinery may render certain codons more efficiently reassigned by one of the two most commonly used orthogonal pairs for genetic code expansion.

## Figures and Tables

**Table 1:** Comparison of the identity of amino acids at 6 positions in the amino acid-binding pocket for *Methanosarcina* sp. Pyl aminoacyl tRNA synthetases capable of activating aromatic amino acids.

Amino Acid Charged by aaRS and Organism of Origin	Amino Acid Residue Position, M. *barkeri* Pyl aaRS ^1^					
	267 (302)	271 (306)	274 (309)	311 (346)	313 (348)	367 (401)
pyrrolysine (wild type), *M. barkeri* [55]	Ala	Tyr	Leu	Asn	Cys	Val
phenylalanine, *M. mazei* [51]	Leu	Met	Leu	Ser	Leu	Val
phenylalanine, *M. mazei* [50]	Ala	Tyr	Leu	Ala	Leu	Val
O-methyl-L-tyrosine, *M. mazei* [45]	Thr	Tyr	Leu	Val	Trp	Leu
4-bromo-L-phenylalanine/4-iodo-L-phenylalanine, *M. mazei* [50]	Ala	Leu	Ser	Ser	Met	Leu
tyrosine, TyrGen1 *M. barkeri*, [this paper]	Lys	Tyr	Met	Ala	Glu	Val

^1^ Residue numbers in parenthesis correspond to the amino acid position in the *M. mazei* Pyl aaRS.

**Table 2:** Impact of sense codon reassignment by the TyrGen2 orthogonal pair on cellular fitness.

Codon Reassigned	Instantaneous Doubling Time (minutes)	Relative Cellular Fitness ^1^
Lys AAG	31.0 ± 0.7	0.926 ± 0.032
Asn AAU	30.2 ± 1.3	0.951 ± 0.049
Glu GAG	33.3 ± 1.7	0.861 ± 0.050
Arg AGG	29.9 ± 0.9	0.959 ± 0.037
Reference system	28.7 ± 0.7	1.00 ± 0.035

^1^ The reference system for 100% cellular fitness combined a non-fluorescent GFP reporter vector with an orthogonal translation machinery vector expressing an inactive version of the Pyl aaRS and the tRNA with a CUA anticodon.

**Table 3:** Discrimination between targeted and non-targeted codons by orthogonal *M. barkeri* tRNAs.

tRNA Anticodon	Targeted Codon	Reassignment Efficiency, TyrGen2 aaRS	Non-Targeted Codon	Reassignment Efficiency, TyrGen2 aaRS	Discrimination Ratio
CUU	AAG	10.9 ± 0.6% ^1^	AAA	B.D. ^2^	99:1
AUU	AAU	2.6 ± 0.3%	AAC	B.D.	≥95:5
CUC	GAG	11.8 ± 0.5%	GAA	0.2 ± 0.04%	98:1
CCU	AGG	65.0 ± 2.7%	AGA	B.D.	>99:1

^1^ Codon reassignment efficiencies reported in this table were measured in *E. coli* SB3930.^2^ B.D. indicates that the measurement was below the detection limit of the in cell assay (0.15%).

## Data Availability

Data are available upon request from the corresponding author for investigators at academic institutions and government research facilities.

## Supplementary Materials

Supplementary materials can be found at https://www.mdpi.com/1422-0067/22/2/895/s1. Supplementary materials include detailed experimental protocols, vector and oligonucleotide sequences, and two figures. Figure S1: Directed evolution workflow, Table S1: Oligonucleotide primers for construction of aaRS gene, Table S2: Oligonucleotide primers for construction of tRNA genes, Table S3: Mutagenic primers for site-directed aaRS library preparation, Figure S2: FACS traces for site-directed amino acid binding pocket aaRS variant library, Table S4: Data summary, EP-PCR round 1, Table S5: Data summary, EP-PCR round 2, Table S6: Number of biological replicates analyzed for each reported codon reassignment efficiency.

## Abbreviations

aaRS	aminoacyl tRNA synthetase
EP-PCR	error-prone polymerase chain reaction
ESI-MS	electrospray ionization mass spectrometry
FACS	fluorescence-activated cell sorting
GFP	green fluorescent protein
IPTG	Isopropyl-beta-D-thiogalactoside
ncAA	non-canonical amino acid
PCR	polymerase chain reaction
