# Supplementary material for: Directed Evolution of the Methanosarcina barkeri Pyrrolysyl tRNA/aminoacyl tRNA Synthetase Pair for Rapid Evaluation of Sense Codon Reassignment Potential
Source: Int J Mol Sci. 2021 Jan 18;22(2):895. doi: 10.3390/ijms22020895 (PMC7830368; doi:10.3390/ijms22020895)
Supplement: Supplementary file 1 [file ijms-22-00895-s001.pdf]

Supplementary Material For:

Directed evolution of the *M. barkeri* pyrrolysine tRNA/aminoacyl tRNA synthetase pair for rapid evaluation of sense codon reassignment potential

David G. Schwark, Margaret A. Schmitt, and John D. Fisk*

Department of Chemistry, University of Colorado Denver,
Campus Box 194, PO Box 173364, Denver, CO 80217

***** Correspondence: john.fisk@ucdenver.edu; Tel.: +01-303-315-7663

TABLE OF CONTENTS

S.1: Cell strains S3
S.2: General reagents and materials S3
Figure S1: Directed evolution workflow S4
S.3: GFP reporter vectors for codon reassignment S5
S.4: TBIO-PCR for synthesis of *M. barkeri* Pyl tRNA and aaRS genes S5
Table S1: Oligonucleotide primers for construction of aaRS gene S6
Table S2: Oligonucleotide primers for construction of tRNA genes S7
S.5: *M. barkeri* tRNA/aaRS pair expression vector backbone details and sequence S7
    S.5.1 Sequence of the *M. barkeri* pyrrolysyl tRNA and $tRNA^{Pyl\text{-}Opt}$ S7
    S.5.2 aaRS promoter sequences S8
    S.5.3 Sequence of the parent *M. barkeri* Pyl aaRS S8
    S.5.4 Sequence of the TyrGen1 aaRS S9
    S.5.5 Sequence of the TyrGen2 aaRS S9
S.6: Preparation of ss dU DNA for site-directed mutagenesis S10
S.7: Preparation of an aaRS library via site-directed Kunkel mutagenesis S11
Table S3: Mutagenic primers for site-directed aaRS library preparation S12
S.8: Preparation of tRNA anticodon variants for evaluation of sense codon reassignment S12
S.9: Fluorescence-activated cell sorting for identification of tyrosine-incorporating aaRS variants S13
Figure S2: FACS traces for site-directed amino acid binding pocket aaRS variant library S14
S.10: Workflow for verification of aaRS activity S15
S.11: Error-prone PCR S16
Table S4: Data summary, EP-PCR round 1 S16
Table S5: Data summary, EP-PCR round 2 S16
S.12: GFP fluorescence-based codon reassignment assay protocol S17
S.13: Calculation of codon reassignment efficiencies S17
Table S6: Number of biological replicates analyzed for each reported codon reassignment efficiency S18
S.14: Expression and purification of Z domain proteins for mass spectrometry S19
    S.14.1 Sequence of pSPEL253 protein expression vectors S19

S.1. Cell strains

DH10B (Invitorgen) F^- *mcr*A Δ(*mrr-hsd*RMS-*mcr*BC) φ80*lac*ZΔM15 Δ*lac*X74 *rec*A1 *end*A1 *ara*D139 Δ(*ara-leu*)7697 *gal*U *gal*K λ^- *rps*L(Str^R) *nup*G

SB3930 (Yale CGSC): λ^-, *ΔhisB463*

CJ236 (New England Biolabs): FΔ*(HindIII)::cat* (Tra^+ Pil^+ Cam^R)/ *ung-1 relA1 dut-1 thi-1 spoT1 mcrA*

S.2 General materials and reagents

All restriction enzymes, DNA polymerases, and T4 kinase were purchased from New England Biolabs and used according to the manufacturer's instructions. ATP was purchased from Fisher (BP413-25) and dNTPs were purchased form New England Biolabs (N0447S). DNA isolation was performed using a Thermo Scientific GeneJET plasmid miniprep kit (K0503) according to the manufacturer's protocols. Some cloning steps and PCR products were purified using a Thermo Scientific GeneJET PCR spin kit (K0701).

LB liquid media (per liter: 10 g tryptone, 5 g yeast extract, 5 g NaCl) and LB agar plates with 15 g/L agar (TEKNova, A7777) were used unless otherwise noted. Isopropyl-beta-D-thiogalactoside (IPTG) was purchased from Gold Bio (I2481C5). Spectinomycin (Enzo Life Science, BML-A281) was used at 50 μg/mL to maintain the vectors harboring the tRNA and aaRS genes. Carbenicillin (PlantMedia, 40310000-2) was used at 50 μg/mL to maintain the vectors harboring the GFP reporter gene. All bacterial cultures were grown at 37 °C unless otherwise noted. All liquid cultures were shaken at 225 rpm unless otherwise noted.

Electrocompetent stocks of all strains were prepared in-house according to the method of Sambrook and Russell (J. Sambrook and D. W. Russell *Molecular cloning: a laboratory manual*. **2001**, Cold Spring Harbor Laboratory press). Typical transformation efficiencies for electrocompetent cells produced in this way are 10^9 cfu/μg of supercoiled DNA. All transformations were recovered in SOC (20 g/L tryptone, 5 g/L yeast extract, 10 mM NaCl, 2.5 mM KCl, 10 mM $MgCl_2$, 20 mM glucose) for 1 hour at 37 °C with shaking prior to transfer to media containing appropriate antibiotics and/or inducers as noted.

All oligonucleotides were purchased from Integrated DNA Technologies (Coralville, Iowa, USA). All DNA sequencing was performed by Genewiz (Plainfield, NJ, USA).

Figure S1: Directed evolution workflow.

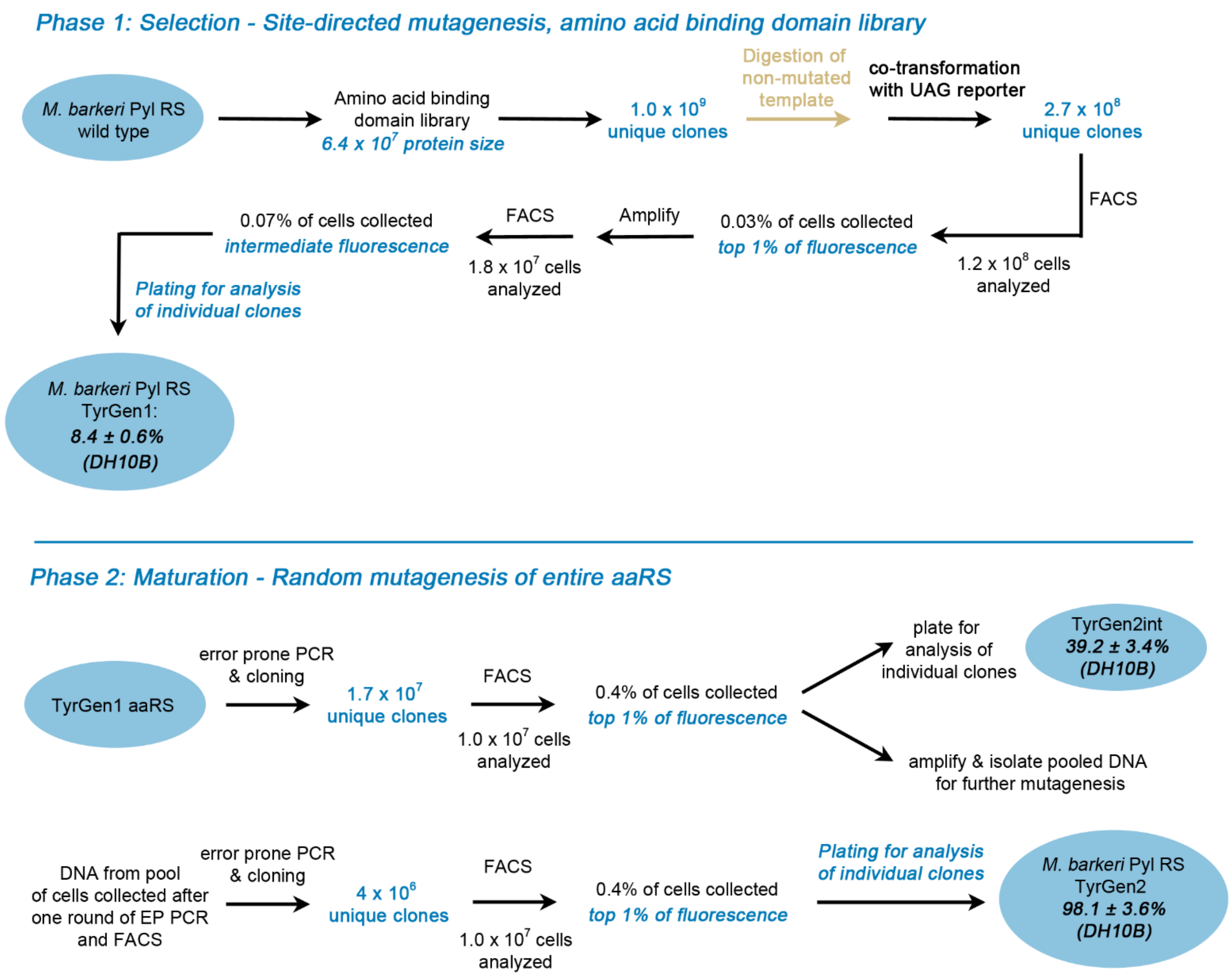

Figure S1. Visual representation of the two phase directed evolution workflow, including key quantifications (e.g. library size, FACS details, codon reassignment efficiencies) for evolution of wild type, pyrrolysine-charging *M. barkeri* aaRS to a variant that charges tyrosine to its cognate tRNA at an efficiency rivaling wild type tRNA/aaRS pairs. Phase 1 describes the evolutionary step of “selection”. Structure and function-guided site-directed mutagenesis resulted in identification of an aaRS that enables its cognate tRNA to decode an amber stop codon as tyrosine with 8.4 ± 0.6% efficiency. In Phase 2, the aaRS is “matured” via random mutagenesis. The resulting aaRS enables its cognate tRNA to decode an amber stop codon as tyrosine with 98.1 ± 3.6%.

S.3 GFP reporter vectors for codon reassignment

All GFP reporter vectors utilized in the evaluation of codon reassignment by the *M. barkeri* tRNA/aaRS pair are those used for evaluation of codon reassignment by the *M. jannaschii* tRNA/aaRS pair.

Full sequence data for the suite of GFP reporter vectors used in this manuscript has been reported previously:

1) W. Biddle, M. A. Schmitt, and J. D. Fisk, Evaluating Sense Codon Reassignment with a Simple Fluorescence Screen, *Biochemistry*, **2015**, *54*, 7355-7364.
2) M. A. Schmitt, W. Biddle, and J. D. Fisk, Mapping the Plasticity of the *Escherichia coli* Genetic Code with Orthogonal Pair-Directed Sense Codon Reassignment. *Biochemistry*, **2018**, *57*, 2762-2774.
3) D. G. Schwark, M. A. Schmitt, W. Biddle, and J. D. Fisk, The Influence of Competing tRNA Abundance on Translation: Quantifying the Efficiency of Sense Codon Reassignment at Rarely Used Codons, *ChemBioChem*, **2020**, *21*, 2274-2286.

S.4 TBIO-PCR synthesis of *M. barkeri* Pyl tRNA and aaRS genes

Both the wild type *M. barkeri* pyrrolysyl tRNA and aaRS (wild type, except for Y349F) genes were prepared using the primers in Table S1 and Table S2 via thermodynamically-balanced inside-out PCR (TBIO-PCR) [Reference: Gao, X. X.; Yo, P.; Keith, A.; Ragan, T. J.; Harris, T. K., Thermodynamically Balanced inside-out (TBIO) PCR-Based Gene Synthesis: A Novel Method of Primer Design for High-Fidelity Assembly of Longer Gene Sequences. *Nucleic Acids Res.* **2003**, *31*, 143e.]

Table S1: Oligonucleotide primers for construction of an *E. coli* codon optimized *M. barkeri* pyrrolysyl aminoacyl tRNA synthetase gene via TBIO-PCR

Primer name	Primer sequence
MbaaRS TBIO 1A	ATGGATAAAAAACCGCTGGATGTGCTGATTAGCG
MbaaRS TBIO 1B	CGCTGGATGTGCTGATTAGCGCGACCGGCCTGTGGATGAGCCGTACCGGCACCC
MbaaRS TBIO 1C	GAGCCGTACCGGCACCCTGCATAAAATCAAACATCATGAAGTGAGCCGCAGCAA
MbaaRS TBIO 1D	CATGAAGTGAGCCGCAGCAAAATCTATATTGAAATGGCGTGCGGCGATCATCTG
MbaaRS TBIO 1E	GCGTGCGGCGATCATCTGGTGGTGAACAACAGCCGTAGCTGCCGTACCGCGCGT
MbaaRS TBIO 1F	CGTTTGCAGGTTTTGCGGTATTTATGATGACGAAACGCACGCGCGGTACGGCAG
MbaaRS TBIO 1G	TGTTGATATCTTCATCGCTCACACGGCAACGTTTGCAGGTTTTGCGGTATTTAT
MbaaRS TBIO 1H	TTTGCTTTCGGTGCTACGGGTCAGAAAGTTGTTGATATCTTCATCGCTCACACG
MbaaRS TBIO 1I	TCGGCGCGCTCACCACACGCACTTTCACGCTGTTTTTGCTTTCGGTGCTACGGG
MbaaRS TBIO 1J	GCACGGCTCACGCTTTTCGGCATCGCTTTTTTCACTTTCGGCGCGCTCACCAC
MbaaRS TBIO 2K	GCCGAAAAGCGTGAGCCGTGCGCCGAAACCGCTGGAAAATA
MbaaRS TBIO 2L	CGCCGAAACCGCTGGAAAATAGCGTGAGCGCGAAAGCGAGCACCAACACCAGCC
MbaaRS TBIO 2M	CGAGCACCAACACCAGCCGTAGCGTTCCGAGCCCGGCGAAAAGCACCCCGAACA
MbaaRS TBIO 2N	GCGAAAAGCACCCCGAACAGCAGCGTTCCGGCGTCTGCGCCGGCACCGAGCCTG
MbaaRS TBIO 2O	GCCGGCACCGAGCCTGACCCGCAGCCAGCTGGATCGTGTGGAAGCGCTGCTGTC
MbaaRS TBIO 2P	GTTTCGCCATGTTCAGGCTAATTTTATCTTCCGGAGACAGCAGCGCTTCCACAC
MbaaRS TBIO 2Q	CACCAGTTCCGGTTCCAGTTCACGAAACGGTTTCGCCATGTTCAGGCTAATTTT
MbaaRS TBIO 2R	ATACAGGCGCTGAAAATCGTTTTTACGACGGGTCACCAGTTCCGGTTCCAGTTC
MbaaRS TBIO 2S	TTGCCCAGATAATCTTCACGATCGTTGGTATACAGGCGCTGAAAATCGTTTTTA
MbaaRS TBIO 2T	CACGTTCCAGTTTGCCCAGATAATCTTCACGATCG
MbaaRS TBIO 3U	ATCTGGGCAAACTGGAACGTGATATCACCA
MbaaRS TBIO 3V	GCAAACTGGAACGTGATATCACCAAATTTTTTGTGGATCGCGGCTTTCTGGAAA
MbaaRS TBIO 3W	GGATCGCGGCTTTCTGGAAATTAAAAGCCCGATTCTGATTCCGGCGGAATATGT
MbaaRS TBIO 3X	GATTCTGATTCCGGCGGAATATGTGGAACGTATGGGCATTAACAACGACACCGA
MbaaRS TBIO 3Y	GGGCATTAACAACGACACCGAACTGAGCAAACAAATTTTCCGCGTGGATAAAAA
MbaaRS TBIO 3Z	CAAACAAATTTTCCGCGTGGATAAAAACCTGTGCCTGCGTCCGATGCTGGCCCC
MbaaRS TBIO 3AA	AATACGATCCAGTTTACGCAGATAGTTATACAGGGTCGGGGCCAGCATCGGACG
MbaaRS TBIO 3AB	CAAAAATTTTGATCGGACCCGGCAGAATACGATCCAGTTTACGCAGATAGTTAT
MbaaRS TBIO 3AC	TCGCTTTCTTTGCGATAGCACGGGCCCACTTCAAAAATTTTGATCGGACCCGGC
MbaaRS TBIO 3AD	CCATGGTGAATTCTTCCAGGTGTTCTTTGCCATCGCTTTCTTTGCGATAGCACG
MbaaRS TBIO 4AE	GTGCAGCCGCTGCCCATTTGGCAAAAGTTAACCATGGTGAATTCTTCCAGGTGT
MbaaRS TBIO 4AF	GCGCTTCCAGGTTTTCACGGGTGCAGCCGCTGCCCA
MbaaRS TBIO 4AG	CGTGAAAACCTGGAAGCGCTGATCAAAGA
MbaaRS TBIO 4AH	CCTGGAAGCGCTGATCAAAGAATTCCTGGATTATCTGGAAATCGACTTCGAAAT
MbaaRS TBIO 4AI	GGATTATCTGGAAATCGACTTCGAAATTGTGGGCGATAGCTGCATGGTGTATGG
MbaaRS TBIO 4AJ	GCGATAGCTGCATGGTGTATGGCGATACCCTGGATATTATGCATGGCGATCTGG
MbaaRS TBIO 4AK	TGGATATTATGCATGGCGATCTGGAACTGAGCAGCGCGGTGGTGGGTCCGGTTA
MbaaRS TBIO 4AL	TCCACGGTTTATCAATGCCCCATTCACGATCCAGGCTAACCGGACCCACCACCG
MbaaRS TBIO 4AM	TCAGCAGACGTTCCAGGCCAAAACCCGCGCCAATCCACGGTTTATCAATGCCCC
MbaaRS TBIO 4AN	CGCACGTTTAATGTTTTTGAAGCCATGCATCACTTTCAGCAGACGTTCCAGGCC
MbaaRS TBIO 4AO	AATGCCGTTATAGTAGCTTTCGCTACGGCTCGCACGTTTAATGTTTTTGAAGCC
MbaaRS TBIO 4AP	TTACAGGTTCGTGCTAATGCCGTTATAGTAGCTTTCGCTA

Table S2: Oligonucleotide primers for construction of the *M. barkeri* pyrrolysyl tRNA and tRNA$^{Pyl\text{-}Opt}$ genes via TBIO-PCR

Primer name	Primer sequence
MbtRNA TBIO A	CCAGGTCTCGAGCATGCAAAAAAGCCTGCTCGTTGAG
MbtRNA TBIO B	AAAAAAGCCTGCTCGTTGAGCAGGCTTTTCGAATTTGGCGGAAACCCCGGG
MbtRNA TBIO C	GGCGGAAACCCCGGGAATCTAACCCGGCTGAACGGATTTAGAGTCCATTCG
MbtRNA TBIO D	GAACGGATTTAGAGTCCATTCGATCTACATGATCAGGTTCCCAATGCGGGG
MbtRNA TBIO E	GATTGACGAGGGCGTATCTGCGCAGTAAGATGCGCCCCGCATTGGGAACCT
MbtRNA TBIO F	TCGCCGATCAAAAGGCATTTTGCTATTAAGGGATTGACGAGGGCGTATCTG
MbtRNA TBIO G	TGCACGGCTAACTAAGCGGCCTGCTGACTTTCTCGCCGATCAAAAGGCATT
MbtRNA TBIO H	ACTAGAGTGCACGGCTAACTAAGCG
MbtRNA^{Opt} TBIO E	GATTGACGAGGGCGTATCTGCGCAGTAAGATGCGCCCCGCATTGGAAACGT
MbtRNA^{Opt} TBIO D	GAACGGATTTAGAGTCCATTCGATCTACATGATCACGTTTCCAATGCGGGG
MbtRNA^{Opt} TBIO C	GGCGGAAACGTGGGGAATCTAACCCCACTGAACGGATTTAGAGTCCATTCG
MbtRNA^{Opt} TBIO B	AAAAAAGCCTGCTCGTTGAGCAGGCTTTTCGAATTTGGCGGAAACGTGGGG

The *Mb* tRNA$^{Pyl\text{-}Opt}$ was constructed in the same manner as the *Mb* tRNA, with the *Mb* tRNA$^{Pyl\text{-}Opt}$ primers indicated above replacing the corresponding primers for the *Mb* tRNA (e.g. MbtRNA TBIO E replaced by MbtRNA^{Opt} TBIO E).

S.5 Vector backbone sequence details for the *M. barkeri* orthogonal translation machinery

The vector backbone from which the *M. barkeri* tRNA/aaRS variants are expressed for evaluation using the fluorescence-based screen is based on the vector used in our previous evaluations of sense codon reassignment by the *M. jannaschii* tRNA/aaRS orthogonal pair. The sequence of the entire vector was reported in W. Biddle, M. A. Schmitt, and J. D. Fisk, Evaluating sense codon reassignment with a simple fluorescence screen, *Biochemistry*, **2015**, *54*, 7355-7364.

Adjustments to the published vector sequence that are reported in this manuscript include the following:

S.5.1 Sequences of the *M. barkeri* pyrrolysyl tRNA and tRNA$^{Pyl\text{-}Opt}$.

The gene sequence for the *M. barkeri* pyrrolysyl tRNA variants ("nnn" is the anticodon, canonical tRNA numbering positions 34-36) is:

5′ – GGG AAC CTG ATC ATG TAG ATC GAA TGG ACT **nnn** AAT CCG TTC AGC CGG GTT AGA TTC CCG GGG TTT CCG CCA – 3′

In all vectors, the anticodon was changed to Watson—Crick base pair with the targeted codon (either amber stop or a sense codon). Unless otherwise noted, all tRNA variants reported in this manuscript have this sequence.

The gene sequence for the *M. barkeri* pyrrolysyl tRNA variant tRNA$^{Pyl\text{-}Opt}$ with anticodon CTA is:

5′ – GGA AAC GTG ATC ATG TAG ATC GAA TGG ACT **CTA** AAT CCG TTC AGT GGG GTT AGA TTC CCC ACG TTT CCG CCA – 3′

TyrGen1 aaRS, under control of the tac promoter, in combination with tRNA$^{Pyl\text{-}Opt}$ reassigned the UAG codon with an efficiency of 18.5 ± 0.8%.

Reference for tRNA$^{Pyl\text{-}Opt}$: C. Fan, H. Xiong, N. M. Reynolds, D. Söll, Rationally evolving tNRA(Pyl) for efficient incorporation of noncanonical amino acids, *Nucleic Acids Research*, **2015**, *43*, e156.

S.5.2 Sequence of the promoter driving the expression of the aaRS.

The reported sequence of the *M. jannaschii* tRNA/aaRS orthogonal pair vector uses the tac promoter to drive expression of the aaRS. Expression of the first generation aaRS variant, TyrGen1, was driven by the lpp promoter. This vector combination reassigned the amber stop codon with an efficiency of 8.4 ± 0.6%.

The sequence of the lpp promoter is:

5′- CCA TCA AAA AAA TAT TCT CAA CAT AAA AAA CTT TGT GTA ATA CTT GTA ACG CT -3′

The first adjustment made to the orthogonal translation machinery vector to improve amber stop codon reassignment was to change the promoter sequence from lpp to tac. The sequence of the tac promoter is that reported in Biddle *Biochemistry* 2015. This vector combination reassigned the amber stop codon with an efficiency of 19.3 ± 1.8%.

The sequence of the tac promoter is:

5′ – TGA CAA TTA ATC ATC GGC TCG TAT AAT GT -3′

S.5.3 Sequence of the parent aaRS:

Protein sequence: *M. barkeri* pyrrolysyl aminoacyl tRNA synthetase

MDKKPLDVLISATGLWMSRTGTLHKIKHHEVSRSKIYIEMACGDHLVVNNSRSCRTARAFRHHKYRKTCKRCRVSDEDINNFLTR

STESKNSVKVRVVSAPKVKKAMPKSVSRAPKPLENSVSAKASTNTSRSVPSPAKSTPNSSVPASAPAPSLTRSQLDRVEALLSPE

DKISLNMAKPFRELEPELVTRRKNDFQRLYTNDREDYLGKLERDITKFFVDRGFLEIKSPILIPAEYVERMGINNDTELSKQIFR

VDKNLCLRPMLAPTLYNYLRKLDRILPGPIKIFEVGPCYRKESDGKEHLEEFTMVNFCQMGSGCTRENLEALIKEFLDYLEIDFE

IVGDSCMV**F**GDTLDIMHGDLELSSAVVGPVSLDREWGIDKPWIGAGFGLERLLKVMHGFKNIKRASRSESYYNGISTNL*

The amino acid in bold (F349) and highlighted in green was a fixed starting mutation. The wild type *M. barkeri* PylRS has a tyrosine at position 349. Takimoto and co-workers also had Y349F in their starting material as this mutation was shown to increase aminoacylation efficiency. Position 349 remained Phe in their identified aaRS for incorporation of O-methyl tyrosine. Reference: J. K. Takimoto, N. Dellas, J. P. Noel, L. Wang, Stereochemical basis for engineered pyrrolysyl-tRNA synthetase and the efficient in vivo incorporation of structurally divergent non-native amino acids, *ACS Chem Biol*, **2011, *6*, 733-743.

S.5.4 Sequence of TyrGen1, the first generation evolved tyrosine-incorporating aaRS:

DNA sequence: 5′ – ATGGATAAAAAACCGCTGGATGTGCTGATTAGCGCGACCGGCCTGTGGATGAGCCGTACCGGCACC
CTGCATAAAATCAAACATCATGAAGTGAGCCGCAGCAAAATCTATATTGAAATGGCGTGCGGCGATCATCTGGTGGTGAACAACA
GCCGTAGCTGCCGTACCGCGCGTGCGTTTCGTCATCATAAATACCGCAAAACCTGCAAACGTTGCCGTGTGAGCGATGAAGATAT
CAACAACTTTCTGACCCGTAGCACCGAAAGCAAAAACAGCGTGAAAGTGCGTGTGGTGAGCGCGCCGAAAGTGAAAAAAGCGATG
CCGAAAAGCGTGAGCCGTGCGCCGAAACCGCTGGAAAATAGCGTGAGCGCGAAAGCGAGCACCAACACCAGCCGTAGCGTTCCGA
GCCCGGCGAAAAGCACCCCGAACAGCAGCGTTCCGGCGTCTGCGCCGGCACCGAGCCTGACCCGCAGCCAGCTGGATCGTGTGGA
AGCGCTGCTGTCTCCGGAAGATAAAATTAGCCTGAACATGGCGAAACCGTTTCGTGAACTGGAACCGGAACTGGTGACCCGTCGT
AAAAACGATTTTCAGCGCCTGTATACCAACGATCGTGAAGATTATCTGGGCAAACTGGAACGTGATATCACCAAATTTTTTGTGG
ATCGCGGCTTTCTGGAAATTAAAAGCCCGATTCTGATTCCGGCGGAATATGTGGAACGTATGGGCATTAACAACGACACCGAACT
GAGCAAACAAATTTTCCGCGTGGATAAAAACCTGTGCCTGCGTCCGATGCTGAAGCCGACCCTGTATAACTATATGCGTAAACTG
GATCGTATTCTGCCGGGTCCGATCAAAATTTTTGAAGTGGGCCCGTGCTATCGCAAAGAAAGCGATGGCAAAGAACACCTGGAAG
AATTTACCATGGTTGCTTTTGAGCAAATGGGCAGCGGCTGCACCCGTGAAAACCTGGAAGCGCTGATCAAAGAATTTCTGGATTA
TCTGGAAATCGACTTCGAAATTGTGGGCGATAGCTGCATGGTGTTTGGCGATACCCTGGATATTATGCATGGCGATCTGGAACTG
AGCAGCGCGGTGGTTGGTCCGGTTAGCCTGGATCGTGAATGGGGCATTGATAAACCGTGGATTGGCGCGGGTTTTGGCCTGGAAC
GTCTGCTGAAAGTGATGCATGGCTTCAAAAACATTAAACGTGCGAGCCGTAGTGAAAGCTACTATAACGGCATTAGCACGAACCT
GTAA – 3′

Protein sequence: The 6 amino acid residues highlighted in blue and in boldface are the 6 amino acid positions which were varied using the NNK codon during library generation. Phe349, a fixed mutation relative to the wild type *M. barkeri* PylRS and discussed above, remains in bold for ease of visualization.

MDKKPLDVLISATGLWMSRTGTLHKIKHHEVSRSKIYIEMACGDHLVVNNSRSCRTARAFRHHKYRKTCKRCRVSDEDINNFLTR
STESKNSVKVRVVSAPKVKKAMPKSVSRAPKPLENSVSAKASTNTSRSVPSPAKSTPNSSVPASAPAPSLTRSQLDRVEALLSPE
DKISLNMAKPFRELEPELVTRRKNDFQRLYTNDREDYLGKLERDITKFFVDRGFLEIKSPILIPAEYVERMGINNDTELSKQIFR
VDKNLCLRPML**K**PTL**Y**NYM**R**KLDRILPGPIKIFEVGPCYRKESDGKEHLEEFTMV**A**F**E**QMGSGCTRENLEALIKEFLDYLEIDFE
IVGDSCMV**F**GDTLDIMHGDLELSSAV**V**GPVSLDREWGIDKPWIGAGFGLERLLKVMHGFKNIKRASRSESYYNGISTNL*

S.5.5 Sequence of TyrGen2, the second generation improved evolved tyrosine-incorporating aaRS:

DNA sequence 5′ – ATGGATAAAAAACCGCTGGATGTGCTGATTAGCGCGACCGGCCTGTGGATGAGCCGTTCCGGCACCC
TGCATAAAATCAAACATCATGAAGTGAGCCGCAGCAAAATCTATATTGAAATGGCGTGCGGCGATCATCTGGTGGTGAACAACAG
CCGTAGCTGCCGTACCGCGCGTGCGTTTCGTCATCATAAATACCGCAAAACCTGCAAACGTTGCCGTGTGAGCGATGAAGATATC
AACAACTTTCTGACCCGTAGCACCGAAAGCAAAAACAGCGTGAAAGTGCGTGTGGTGAGCGCGCCGAAAGTGAAAAAAGCGATGC
CGAAAAGCGTGAGCCGTGCGCCGAAACCGCTGGAAAATTGCGTGAGCGCGAGAGCGAGCACCAACACCAGCCGTAGCGTTCCGAG
CCCGGCGAAAAGCACCCCGAACAGCAGCGTTCCGGCGTCTGCGCCGGCACCGAGCCTGACCCGCAGCCAGCTGGATCGTGTGGAA
GCGCTGCTGTCTCCGGAAGATAAAATTAGCCTGAACATGGCGAAACCGTTTCGTGAACTGGAACCGGAACTGGTGACCCGTCGTA
AAAACGATTTTCAGCGCCTGTATACCAACGATCGTGAAGATTATCTGGGCAAACTGGAACGTGATATCACCAAATTTTTTGTGGA
TCGCGGCTTTCTGGAAATTAAAAGCCCGATTCTGATTCCGGCGGAATATGTGGAACGTATGGGCATTAACAACGACACCGAACTG
AGCAAACAAATTTTCCGCGTGGATAAAAACCTGTGCCTGCGTCCGACGCTGAAGCCGACCCTGTATAACTATATGCGTAAACTGG
ATCGTATTCTGCCGGGTCCGATCAAAATTTTTGAAGTGGGCCCGTGCTATCGCAAAGAAAGCGATGGCAAAGAACACCTGGAAGA

ATTTACCATGGTTGCTTTTGAGCAAATGGGCAGCGGCTGCACCCGTGAAAACCTGGAAGCGCTGATCAAAGAATTTCTGGATTAT
CTGGAAATCGACTTCGAAATTGTGGGCGATAGCTGCATGGTGTTTGGCGATACCCTGGATATTATGCATGGCGATCTGGAACTGA
GCAGCGCGGTGGTTGGCCCGGTTAGCCTGGATCGTGAATGGGGCATTGATAAACCGTGGATTGGCGCGGGTTTTGGCCTGGAACG
TCTGCTGAAAGTGATGCATGGCTTCAAAAACATTAAACGTGCGAGCCGTAGCGTAAGCTACTATAACGGCATTAGCACGAACCTG
TAA - 3′

Protein sequence: The 5 amino acid residues highlighted in yellow and in boldface are those that mutated from the first generation aaRS via non-targeted error prone PCR mutagenesis and high-throughput analysis. The 6 amino acid mutations identified from the first targeted library did not change in the second-generation aaRS. These positions remain in bold. Phe 349, a fixed mutation relative to the wild type *M. barkeri* PylRS and discussed above, remains in bold for ease of visualization.

MDKKPLDVLISATGLWMSR**S**GTLHKIKHHEVSRSKIYIEMACGDHLVVNNSRSCRTARAFRHHKYRKTCKRCRVSDEDINNFLTR
STESKNSVKVRVVSAPKVKKAMPKSVSRAPKPLEN**C**VSA**R**ASTNTSRSVPSPAKSTPNSSVPASAPAPSLTRSQLDRVEALLSPE
DKISLNMAKPFRELEPELVTRRKNDFQRLYTNDREDYLGKLERDITKFFVDRGFLEIKSPILIPAEYVERMGINNDTELSKQIFR
VDKNLCLRP**T**L**K**PTL**Y**NY**M**RKLDRILPGPIKIFEVGPCYRKESDGKEHLEEFTMV**A**F**E**QMGSGCTRENLEALIKEFLDYLEIDFE
IVGDSCMV**F**GDTLDIMHGDLELSSAV**V**GPVSLDREWGIDKPWIGAGFGLERLLKVMHGFKNIKRASRS**V**SYYNGISTNL*

S.6. Preparation of ss dU DNA for site-directed Kunkel mutagenesis

The original *M. barkeri* aaRS variant library and *M. barkeri* tRNA anticodon variants were produced with Kunkel mutagenesis using a method adapted from Sidhu and Weiss (S. Sidhu and G. Weiss *Phage display: a practical approach* **2004** (Clackson, T. and Lowman, H.B., Ed.). Oxford University Press, USA). This mutagenic strategy relies upon preparation of single-stranded template DNA enriched in dU content. DNA is prepared in a cell strain lacking two of the enzymes responsible for editing deoxyuridine from newly synthesized DNA. Following annealing of mutagenic primers and extension of the template DNA in the absence of deoxyuridine, the double-stranded DNA is transformed into cells that have the enzymes responsible for editing dU out of DNA. The non-mutated, template strand of DNA is degraded, and the mutated strand is replicated and transcribed.

Briefly, 4 mL cultures of CJ236 cells harboring the phagemid to be mutated were grown to an OD_{600} of 0.5 and infected with M13KO7 helper phage at a multiplicity of infection of 10:1. The infected culture was transferred into 125 mL of LB media with 5 μg/mL chloramphenicol, appropriate antibiotic to maintain the phagemid, and 0.25 μg/mL uridine. Cultures were grown overnight at 30 °C. Cells were pelleted at 17,000 x*g* at 4 °C for 20 minutes in a Sorvall RC 6+ with a Thermo FIBERLite F14-6x250y rotor. Phage particles were isolated by decanting the supernatant from the pelleted cells into 1/5th volume of 20% 8,000 molecular weight polyethylene glycol and 2.5M NaCl in water. Solutions were incubated on ice for at least 2 hours. Phage particles were isolated by pelleting at 17,000 x*g* at 4 °C for 20 minutes. The supernatant was decanted,

and the phage pellet was spun for an additional minute to collect remaining supernatant, which was then removed. The phage pellet was resuspended in 1.5 mL of phosphate buffered saline, pH = 7.4. Insoluble material was pelleted out of the phage solution at 17,000 x*g* for 5 minutes. Single-stranded, uridine-enriched DNA (ss dU DNA) was isolated from phage using a Qiagen M13 spin kit.

S.7. Preparation of the site-directed aaRS library

Three mutagenic primers (Table S3) were phosphorylated for 1.5 hours at 37 °C using T4 polynucleotide kinase. Phosphorylated primers were annealed to 5 μg of single-stranded template DNA at a 3:1 molar ratio by incubating at 90 °C for 2 minutes, 70 °C for 20 seconds, followed by a decreasing temperature ramp of 1°C every 20 seconds until the temperature reached 20 °C. The reaction was held at 20 °C for 2 minutes. The annealed mixture was extended using T7 DNA polymerase in the presence of T4 DNA ligase and 670 μM ATP and 330 μM dNTPs (each) at room temperature overnight.

The crude mutagenesis reaction was purified using a PCR spin kit column. The entirety of the eluted purified DNA (55 μL) was transformed into electrocompetent *E. coli* DH10B to degrade the ss dU DNA template. Transformed cells were allowed to recover in 6 mL of SOC without antibiotics for 1 hour at 37 °C. Following recovery, a small aliquot was removed for plating onto LB agar/spectinomycin to determine the size of the library. 1 x 10^8 unique clones were generated.

The remainder of the SOC recovery media was diluted into LB with spectinomycin (160 mL total volume) and grown for approximately 2 doublings based on the starting OD_{600} (2 hours and 20 minutes). 15.3 μg of plasmid DNA was purified from the culture using 3 miniprep columns and digested with KpnI-HF (NEB) for 2 hours at 37 °C to remove nonmutated and partially mutated DNA. The digested DNA was purified using 2 PCR spin columns. The contents of each column were eluted using 55 μL of sterile water for a total of 110 μL of eluted DNA.

Purified, digested DNA was transformed into electrocompetent *E. coli* DH10B already harboring the GFP reporter plasmid with a UAG amber codon at position 66. Transformations were recovered in a total of 50 mL SOC for 1 hour. A small aliquot of the recovery was plated to determine transformation efficiency. 2.7 x 10^8 unique transformants were acquired. 19/23 clones characterized by cPCR and sequencing were unique library members.

The remainder of the SOC recovery media was diluted into LB media with appropriate antibiotics and 1 mM IPTG to induce expression of both the aaRS variants and the GFP reporter (600 mL total volume) and grown overnight. The following morning, the library aliquoted into volumes containing 0.3 ODs and frozen at -80 °C in 35% glycerol.

Table S3: Mutagenic primers for site-directed aaRS library

Mutagenic primers for site-directed aaRS library preparation	
**MbaaRS Library 1**	GGCAGAATACGATCCAGTTTACGCAGATAGTTATACAGGGTCGGGGCCAGCATCGGACGCAGG
**MbaaRS Library 2**	GCCGCTGCCCATTTGGCAAAAGTTAACCATGGTAAATTCTTCCAGGTGTTCTTTGCC
**MbaaRS Library 3**	CCAGGCTAACCGGACCCACCACCGCGCTGCTCAGTTC

S.8. Preparation of the tRNA anticodon variants for sense codon reassignment

Similar to preparation of the aaRS variant library, Kunkel mutagenesis was used to prepare anticodon variants of the *M. barkeri* tRNA. Briefly, a single mutagenic primer was phosphorylated for 1.5 hours at 37 °C using T4 polynucleotide kinase. Phosphorylated primers were annealed to 500 ng of single-stranded template DNA at a 3:1 molar ratio by incubating at 90 °C for 2 minutes, 53 °C for 3 minutes, and 25 °C for 5 minutes. The annealed mixture was extended using T7 DNA polymerase in the presence of T4 DNA ligase and 670 µM ATP and 330 µM dNTPs (each) at room temperature overnight. The total reaction volume was typically 30 µL. 2µL of each crude mutagenesis reaction was transformed into electrocompetent *E. coli* DH10B and recovered for 1 hour in 1 mL SOC. This procedure typically yields 10^5 transformants with 40-80% mutation efficiency. tRNA variants were easily identified by digesting crude colony PCR products with XhoI. The absence of an XhoI site indicates successful mutation to the desired anticodon. All variants were confirmed by sequencing (Genewiz, LLC).

tRNA variants were prepared using a mutagenic primer having the following basic sequence:

5′ – ACC TGA TCA TGT AGA TCG AAT GGA CT**n** **nn**A ATC CGT TCA GCC GGG TTA G – 3′
where "nnn" specifies the desired anticodon.

Primers for cPCR and sequencing of tRNA anticodon variants:

Fwd: 5′- CCC TTT AGG GTT CCG ATT TAG TGC -3′

Rev: 5′- CAT GGG GTC AGG TGG GAC -3′

Product is 2023 nt long. Cutting the crude product of PCR using the starting tRNA vector XhoI produces two pieces: 469 nt and 1554 nt. PCR products from desired, anticodon altered tRNAs are uncut.

S.9 Fluorescence-activated cell sorting (FACS) for identification of tyrosine-incorporating *M. barkeri* aaRS variants

Tubes of cells harboring the aaRS library and the UAG codon GFP reporter that had been stored at -80 °C in 35% glycerol were thawed on ice and centrifuged at 8,000 xg for 5 minutes at room temperature. The supernatant was removed via pipette, and 1 mL sterile 0.9% aqueous NaCl was added to each tube. Cells were resuspended via gentle pipetting and returned to ice.

FACS screening for identification of the TyrGen1 aaRS variant was performed on a Dako-Cytomation MoFlo Legacy instrument using the 488 nm laser line with a 530/40 nm band-pass filter in front and the 70 μm flow cell tip. For all runs, cells were initially gated on forward and side scatter to remove clumps and non-cell particles. Cells were then gated on GFP fluorescence and sorting was performed on the purify 1,2 mode at approximately 21,000 events/second. Following sorting, collected cells were diluted directly into LB with appropriate antibiotics and IPTG to a final volume of 6 mL and grown overnight. The next day, aliquots of the saturated culture containing 0.3 ODs were stored at -80 °C in 35% glycerol for future use (e.g. additional FACS sorting).

Controls for FACS included wild type GFP (DH10B co-transformed with the GFP reporter with a Tyr codon at position 66 and the *M. barkeri* Pyl tRNA/aaRS vector expressing the aaRS template for library synthesis) as well as a non-fluorescent control (DH10B co-transformed with a non-fluorescent GFP reporter (UAG at position 66) and the *M. barkeri* Pyl tRNA/aaRS vector expressing the aaRS template for library synthesis). Prior to library evaluation, approximately 1.2 x 10^6 cells from each sample were analyzed to map the two control populations. Aliquots for flow cytometry were prepared in a fashion identical to that described for cells harboring library members.

Library sort #1: 1.2 x 10^8 cells were analyzed and 3.1 x 10^4 cells (~0.02% of total cells examined) with fluorescence representing the top 1% of clones were collected into LB. Collected cells were those with greater than 12.0 arbitrary fluorescence units. Fluorescence level selected in order to collect the top 1% of clones. Cells were amplified as described above.

Library sort #2: 1.8 x 10^7 amplified from the first sort were analyzed and 1.3 x 10^4 cells (~0.07% of total cells examined) were collected into LB. Collected cells were those with fluorescence greater than the tail observed for the non-fluorescent control population (15.6 arbitrary fluorescence units) AND less than 270 arbitrary fluorescence units. Cells within the highest fluorescent population were suspected to be revertants to wild-type GFP, a phenomenon we have previously observed in high throughput analysis of fluorescent gain of function libraries. Cells were amplified and preserved as described above. A small portion of the amplified cells were also plated for analysis of individual clones.

Figure S2: FACS traces for screening of tyrosine-charging aaRS from amino acid binding pocket targeted library

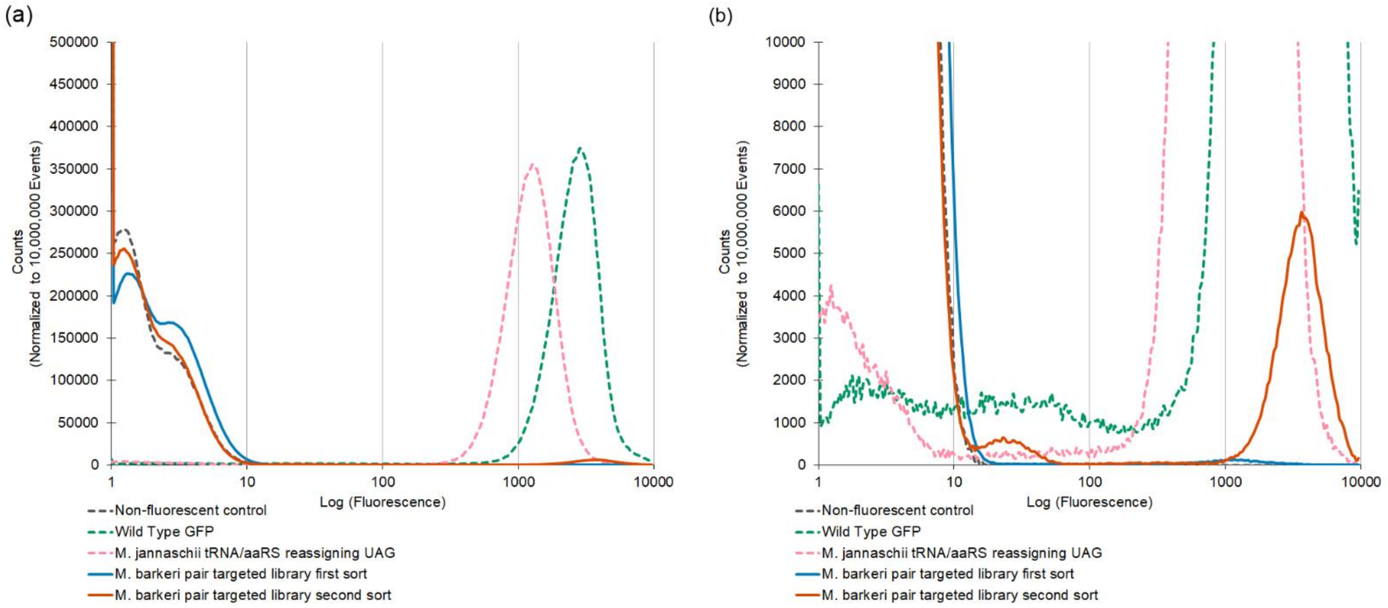

Figure S2. FACS traces for screening of the site-directed mutagenesis library of amino acid binding pocket aaRS variants. (a) full y-axis, to 500,000 counts (b) y-axis restricted to a maximum of 10,000 counts for observation of minor populations. Profiles representing the following control populations are shown: non-fluorescent cells as a control profile (grey, dashed line); cells expressing wild type GFP (green, dashed line); and cells expressing the *M. jannaschii* tRNA/aaRS pair (CUA anticodon) and the GFP reporter with UAG at position 66 (pink, dashed line). The first FACS screening of the library (blue, solid line) reveals a slight population shift with a small amount of fluorescence. Cells with fluorescence in the top 1% of the overall population were collected and amplified. The second FACS sort of the library (orange, solid line) is enriched in the population of cells with some fluorescence. A second, separate population of highly fluorescent cells with a profile similar to that of wild type GFP was observed. These cells were collected separately from the other fluorescent population to segregate cells likely containing GFP fluorophore revertants.

S.10. Workflow for verification of aaRS variant activity

Analysis of individual clones using the *in vivo* fluorescence-based screen precludes the inclusion of biological replicates. Following characterization of the performance of an individual clone, several additional evaluations are undertaken to confirm system behavior.

First, vector DNA is isolated from the individual clone. The DNA includes both the GFP reporter and the orthogonal translation machinery vectors. Restriction enzymes with recognition sites unique to the GFP reporter vector are used to separate the two vectors. Following digestion, the reaction is transformed into electrocompetent *E. coli* DH10B and plated onto LB agar containing either the antibiotic to which resistance is conferred by the orthogonal translation machinery vector (spectinomycin) or both spectinomycin and carbenicillin (the antibiotic upon which cells harboring intact GFP reporter vector can grow) to confirm the successful digestion of the GFP reporter vector. A single colony is grown up in ~8 mL LB/Spectinomycin, and the DNA is isolated and sequenced.

Isolated DNA is co-transformed with the appropriate GFP reporter vector, and multiple colonies are evaluated in the fluorescence-based screen. In this case, the GFP reporter vector DNA has not been shuttled through rounds of high throughput screening and amplification. This additional step not only provides an opportunity to evaluate biological replicates of a given system but also reveals false positives from the initial single colony evaluation that were the result of a fluorophore sequence revertant of the GFP reporter gene (UAG amber stop to UAU or UAC for tyrosine).

Following confirmation that apparent enzyme activity was not the result of an undesired mutation within the reporter vector, a final set of evaluations was undertaken to confirm the activity of the aaRS. Just as multiple rounds of high throughput functional sorting and amplification could inadvertently result in amplification of clones with mutations to the GFP reporter vector, undesired mutations could also result in apparent improved activity of the translational machinery. Mutations that increase gene expression would lead to the false impression that an aaRS is more effective than it really is.

In order to confirm that an identified aaRS sequence enables reassignment of a given codon in a particular system with the reported efficiency, the aaRS gene was PCR amplified out of the isolated DNA and recloned into the backbone vector. Again, the vector DNA used in the cloning reaction had not been through rounds screening and amplification and is not expected to have undesired mutations. The cloned DNA is isolated and sequenced to confirm that the sequence has not changed. This DNA is co-transformed with a GFP reporter vector with the codon of interest specifying the fluorophore tyrosine and evaluated in the fluorescence-based screen. Only after demonstrating consistent codon reassignment efficiency in each of these tests is an aaRS considered active.

S.11. Error prone PCR

PCR conditions known to lower the fidelity of Taq polymerase and an unequal concentration of nucleotides were used to generate a library of TyrGen1 aminoacyl tRNA synthetase variants with random mutations throughout the entire sequence. TyrGen1 was amplified with the intention to introduce 1-4 mutations per 1000 nucleotides. 10 ng of template DNA was included in a reaction with 1.0 mM dCTP and dTTP and 0.2 mM dATP and dGTP. Standard Taq buffer (NEB) with a final concentration of 7 mM $MgCl_2$ was used as the reaction buffer. The final concentration of each amplification primer in the final reaction was 0.4 µM.

Following initial denaturation, 25 cycles of denaturation, annealing, and extension were performed. To enhance the permissivity of Taq polymerase, the extension temperature was increased to 72 °C (as opposed to the optimal Taq extension temperature of 68 °C).

The purified EP-PCR products were digested for ligation into the *M. barkeri* orthogonal translation machinery backbone vector. Ligated products were transformed into DH10B cells harboring the UAG stop codon GFP reporter vector. A small portion of the recovery media was plated to determine transformation efficiency and mutation frequency. The remainder of the transformed cells were transferred to media containing IPTG and appropriate antibiotics and grown overnight. Cells were prepared for FACS analysis as described. FACS was performed as described previously, except that a BD FACSAria III instrument (BD Biosciences, San Jose, CA) was used. Relevant details (number of cells sorted, etc) are given below:

Table S4: Details for generation and screening of EP-PCR library 1

**Error-prone PCR Round 1**	
**Template**	TyrGen1 aaRS
**Unique transformants**	1.7 x 10^7
**Average mutation frequency**	2.2/1000 nucleotides
**Cells screened by FACS**	1.0 x 10^7
**Cells collected**	Top 1% of fluorescence, 0.4% of the population

Table S5: Details for generation and screening of EP-PCR library 2

**Error-prone PCR Round 2**	
**Template**	Pooled DNA from amplification of cells collected after FACS analysis of EP-PCR round 1 library
**Unique transformants**	4.0 x 10^6
**Average mutation frequency**	2.2/1000 nucleotides
**Cells screened by FACS**	1.0 x 10^7
**Cells collected**	Top 1% of fluorescence, 0.4% of the population

S.12. GFP fluorescence-based sense codon reassignment assays

Superfolder green fluorescent protein (GFP) reporter plasmids were co-transformed with vectors expressing the modified orthogonal translational components into either *E. coli* DH10B or SB3930. After overnight growth, colonies were picked into 200 μL LB media in a 96 well plate. Cells were grown to at least mid-log phase (usually 8-10 hours) with shaking at 37 °C. Cells were diluted 10-fold into LB media with antibiotics to maintain the plasmids and 1 mM IPTG for induction of both the aaRS and GFP. Assays were performed in a Fluorotrac 200 clear bottom 96 well plate (Greiner 655096) and monitored in a BioTek Synergy H1 or BioTek Synergy Neo 2S plate reader at 37 °C with continuous double orbital shaking. The optical density (OD600) and fluorescence of each well was measured every 15 minutes for at least 15 hours; optical density was measured at 600 nm, and fluorescence was measured with an excitation at 485 nm and detection at 515 nm with a 20 nm band pass.

S.13 Calculation of sense codon reassignment efficiency from optical density and fluorescence readings

For each biological replicate, the relative fluorescence (corrected fluorescence per OD) was calculated for each of the 16 data points gathered between 8 and 12 hours after induction of GFP and the aaRS with IPTG. The 16 relative fluorescence values were averaged to determine the RFU for each sample. That RFU is divided by the average RFU for all biological replicates of the 100% reassigning fluorescence control (wild type GFP) to determine the reassignment efficiency. Sense codon reassignment efficiency for each tRNA anticodon/GFP codon variant pair was calculated by averaging the reassignment efficiency for at least six biological replicates. An extremely detailed discussion and workflow for calculating codon reassignment efficiency was provided in the Supporting Information for M. A. Schmitt, W. Biddle, and J. D. Fisk, Mapping the Plasticity of the *Escherichia coli* Genetic Code with Orthogonal Pair-Directed Sense Codon Reassignment. *Biochemistry*, **2018**, *57(19)*, 2762-2774.

Table S6: Number of biological replicates analyzed for each codon reassignment measurement

tRNA anticodon	codon at position 66 in GFP reporter	aaRS variant	other translation machinery details	*E. coli* stratin	reassignment efficiency	number of biological replicates
CUA	UAG	TyrGen1	lpp promoter	DH10B	8.4 ± 0.6%	23
CUA	UAG	TyrGen1	tac promoter	DH10B	19.3 ± 1.8%	44
CUA	UAG	TyrGen1	$tRNA^{Pyl-Opt}$	DH10B	18.5 ± 0.8%	6
CUA	UAG	TyrGen2int	tac promoter	DH10B	39.2 ± 3.4%	15
CUA	UAG	TyrGen2	tac promoter	DH10B	98.1 ± 3.6%	18
CUA	UAG	TyrGen1	tac promoter	SB3930	12.6 ± 0.5%	6
CUA	UAG	TyrGen2	tac promoter	SB3930	65.2 ± 1.7%	78
CUA	UAG	*M. jannaschii*	tac promoter	SB3930	86.0 ± 1.6%	6
AUU	AAU	TyrGen1	tac promoter	SB3930	0.7 ± 0.05%	6
AUU	AAU	TyrGen2	tac promoter	SB3930	2.6 ± 0.3%	12
AUU	AAU	*M. jannaschii*	tac promoter	SB3930	7.2 ± 0.4%	12
AUU	AAC	TyrGen2	tac promoter	SB3930	B.D.	6
CUU	AAG	TyrGen1	tac promoter	SB3930	1.9 ± 0.07%	6
CUU	AAG	TyrGen2	tac promoter	SB3930	10.9 ± 0.6%	12
CUU	AAG	*M. jannaschii*	tac promoter	SB3930	7.5 ± 0.7%	12
CUU	AAA	TyrGen2	tac promoter	SB3930	B.D.	6
CUC	GAG	TyrGen1	tac promoter	SB3930	1.1 ± 0.07%	6
CUC	GAG	TyrGen2	tac promoter	SB3930	11.8 ± 0.5%	12
CUC	GAG	*M. jannaschii*	tac promoter	SB3930	18.9 ± 1.0%	30
CUC	GAA	TyrGen2	tac promoter	SB3930	0.2 ± 0.04%	6
CCU	AGG	TyrGen1	tac promoter	SB3930	13.6 ± 0.6%	6
CCU	AGG	TyrGen2	tac promoter	SB3930	65.0 ± 2.7%	12
CCU	AGG	*M. jannaschii*	tac promoter	SB3930	50.0 ± 4.3%	27
CCU	AGA	TyrGen2	tac promoter	SB3930	B.D.	6

S.14 Expression and purification of Z domain proteins for mass spectrometry analysis

Z domain of protein A reporter plasmids pSPEL253-F5Y (mass control for only tyrosine incorporation) and pSPEL253-F5Amber (test case) were co-transformed into DH10B with vectors containing the orthogonal engineered *M. barkeri* translational components TyrGen1 or TyrGen2. Three isolated colonies of each sample were grown overnight in ~8 mL volume (LB with appropriate antibiotics). Overnight cultures were diluted to a starting OD_{600} of approximately 0.1 in 40 mL pre-warmed LB with antibiotics and IPTG to express the engineered aaRS and the reporter protein. Proteins expression proceeded for 12 hours at 37 °C and 225 rpm. 30 OD's of cells were pelleted at 17,000 x *g*, 4 °C for 30. The supernatant was discarded, and the cell pellets were frozen at -20 °C overnight. The following morning, cell pellets were thawed at room temperature, and 1.8 mL of B-Per lysis reagent (Thermo Scientific 78248, prepared according to manufacturer's instructions) was added to each cell pellet. After 30 minutes at room temperature, the lysed cell culture was clarified by centrifugation at 17,000 x *g*, 4 °C for 1 hour. Clarified lysate for each sample was transferred to a clean tube. 20 µL of each sample was saved for SDS-PAGE analysis.

The Z domain of protein A was then purified from the remaining amount of clarified lysate for each sample using Qiagen Ni-NTA spin columns (Qiagen 31014) according to the manufacturer's instructions. 20 µL from each step in the purification process was saved for SDS-PAGE analysis. Protein bound to each was eluted twice with 2 successive 250 µL volumes of NPI-500 buffer (500 µL total). Protein samples were buffer-exchanged into HPLC-grade water using a 3,000 molecular weight cutoff column according to manufacturer's instructions (Millipore UF500396). The final volume of each protein sample after buffer exchange was 300 µL.

ESI-MS was performed in the Chemistry Instrumentation Facility at Colorado State University. Spectra were deconvoluted using the Maximum Entropy algorithm and MassHunter Software from Agilent Technologies.

S.14.1 DNA sequence for pSPEL253-F5* protein expression vectors

The pSPEL253 Z domain protein expression vector pSPEL253-F5AGG was a generous gift from the laboratory of Tae Hyeon Yoo. (Reference: B. S. Lee, S. Seunggun, J. Y. Jeon, K-S. Jang, B. Y. Lee, S. Choi, T. H. Yoo, Incorporation of Unnatural Amino Acids in Response to the AGG Codon, *ACS Chem Biol*, **2015**, *10*, 1648-1653)

The codon corresponding to the amino acid at position 5 of the Z domain was mutated to either F5Y or F5amber.

The sequence of the Z domain gene is: “nnn” corresponds to the codon whose identity is changed across the suite of vectors.

ATGAGAGGATCGCATCACCATCACCATCACGGATCCATGGCCGTAGACAACAAA**nnn**AACAAAGAACAACA
AAACGCGTTCTATGAGATCTTACATTTACCTAACTTAAACGAAGAACAACGAAACGCCTTCATCCAAAGTT
TAAAAGATGACCCAAGCCAAAGCGCTAACCTTTTAGCAGAAGCTAAAAAGCTAAATGATGCTCAGGCGCCG
TGG

The sequence of the parent vector, pSPEL253-F5AGG is:

CTCGAGAAATCATAAAAAATTTATTTGCTTTGTGAGCGGATAACAATTATAATAGATTCAATTGTGAGCGG
ATAACAATTTCACACAGAATTCATTAAAGAGGAGAAATTAACTATGAGAGGATCGCATCACCATCACCATC
ACGGATCCATGGCCGTAGACAACAAAAGGAACAAAGAACAACAAAACGCGTTCTATGAGATCTTACATTTA
CCTAACTTAAACGAAGAACAACGAAACGCCTTCATCCAAAGTTTAAAAGATGACCCAAGCCAAAGCGCTAA
CCTTTTAGCAGAAGCTAAAAAGCTAAATGATGCTCAGGCGCCGTGGTAAAAGCTTAATTAGCTGAGCTTGG
ACTCCTGTTGATAGATCCAGTAATGACCTCAGAACTCCATCTGGATTTGTTCAGAACGCTCGGTTGCCGCC
GGGCGTTTTTTATTGGTGAGAATCCAAGCTAGCTTGGCGAGATTTTCAGGAGCTAAGGAAGCTAAAATGGA
GAAAAAAATCACTGGATATACCACCGTTGATATATCCCAATGGCATCGTAAAGAACATTTTGAGGCATTTC
AGTCAGTTGCTCAATGTACCTATAACCAGACCGTTCAGCTGGATATTACGGCCTTTTTAAAGACCGTAAAG
AAAAATAAGCACAAGTTTTATCCGGCCTTTATTCACATTCTTGCCCGCCTGATGAATGCTCATCCGGAATT
TCGTATGGCAATGAAAGACGGTGAGCTGGTGATATGGGATAGTGTTCACCCTTGTTACACCGTTTTCCATG
AGCAAACTGAAACGTTTTCATCGCTCTGGAGTGAATACCACGACGATTTCCGGCAGTTTCTACACATATAT
TCGCAAGATGTGGCGTGTTACGGTGAAAACCTGGCCTATTTCCCTAAAGGGTTTATTGAGAATATGTTTTT
CGTCTCAGCCAATCCCTGGGTGAGTTTCACCAGTTTTGATTTAAACGTGGCCAATATGGACAACTTCTTCG
CCCCCGTTTTCACCATGGGCAAATATTATACGCAAGGCGACAAGGTGCTGATGCCGCTGGCGATTCAGGTT
CATCATGCCGTTTGTGATGGCTTCCATGTCGGCAGAATGCTTAATGAATTACAACAGTACTGCGATGAGTG
GCAGGGCGGGGCGTAATTTTTTTAAGGCAGTTATTGGTGCCCTTAAACGCCTGGGGTAATGACTCTCTAGC
TTGAGGCATCAAATAAAACGAAAGGCTCAGTCGAAAGACTGGGCCTTTCGTTTTATCTGTTGTTTGTCGGT
GAACGCTCTCCTGAGTAGGACAAATCCGCCCTCTAGATTACGTGCAGTCGATGATAAGCTGTCAAACATGA
GAATTGTGCCTAATGAGTGAGCTAACTTACATTAATTGCGTTGCGCTCACTGCCCGCTTTCCAGTCGGGAA
ACCTGTCGTGCCAGCTGCATTAATGAATCGGCCAACGCGCGGGGAGAGGCGGTTTGCGTATTGGGCGCCAG
GGTGGTTTTTCTTTTCACCAGTGAGACGGGCAACAGCTGATTGCCCTTCACCGCCTGGCCCTGAGAGAGTT
GCAGCAAGCGGTCCACGCTGGTTTGCCCCAGCAGGCGAAAATCCTGTTTGATGGTGGTTAACGGCGGGATA
TAACATGAGCTGTCTTCGGTATCGTCGTATCCCACTACCGAGATATCCGCACCAACGCGCAGCCCGGACTC
GGTAATGGCGCGCATTGCGCCCAGCGCCATCTGATCGTTGGCAACCAGCATCGCAGTGGGAACGATGCCCT
CATTCAGCATTTGCATGGTTTGTTGAAAACCGGACATGGCACTCCAGTCGCCTTCCCGTTCCGCTATCGGC
TGAATTTGATTGCGAGTGAGATATTTATGCCAGCCAGCCAGACGCAGACGCGCCGAGACAGAACTTAATGG
GCCCGCTAACAGCGCGATTTGCTGGTGACCCAATGCGACCAGATGCTCCACGCCCAGTCGCGTACCGTCTT
CATGGGAGAAAATAATACTGTTGATGGGTGTCTGGTCAGAGACATCAAGAAATAACGCCGGAACATTAGTG
CAGGCAGCTTCCACAGCAATGGCATCCTGGTCATCCAGCGGATAGTTAATGATCAGCCCACTGACGCGTTG
CGCGAGAAGATTGTGCACCGCCGCTTTACAGGCTTCGACGCCGCTTCGTTCTACCATCGACACCACCACGC
TGGCACCCAGTTGATCGGCGCGAGATTTAATCGCCGCGACAATTTGCGACGGCGCGTGCAGGGCCAGACTG
GAGGTGGCAACGCCAATCAGCAACGACTGTTTGCCCGCCAGTTGTTGTGCCACGCGGTTGGGAATGTAATT
CAGCTCCGCCATCGCCGCTTCCACTTTTTCCCGCGTTTTCGCAGAAACGTGGCTGGCCTGGTTCACCACGC
GGGAAACGGTCTGATAAGAGACACCGGCATACTCTGCGACATCGTATAACGTTACTGGTTTCACATTCACC
ACCCTGAATTGACTCTCTTCCGGGCGCTATCATGCCATACCGCGAAAGGTTTTGCACCATTCGATGGTGTC
GGAATTTCGGGCAGCGTTGGGTCCTGGCCACGGGTGCGCATGATCTAGAGCTGCCTCGCGCGTTTCGGTGA
TGACGGTGAAAACCTCTGACACATGCAGCTCCCGGAGACGGTCACAGCTTGTCTGTAAGCGGATGCCGGGA
GCAGACAAGCCCGTCAGGGCGCGTCAGCGGGTGTTGGCGGGTGTCGGGGCGCAGCCATGACCCAGTCACGT
AGCGATAGCGGAGTGTATACTGGCTTAACTATGCGGCATCAGAGCAGATTGTACTGAGAGTGCACCATATG

CGGTGTGAAATACCGCACAGATGCGTAAGGAGAAAATACCGCATCAGGCGCTCTTCCGCTTCCTCGCTCAC
TGACTCGCTGCGCTCGGTCGTTCGGCTGCGGCGAGCGGTATCAGCTCACTCAAAGGCGGTAATACGGTTAT
CCACAGAATCAGGGGATAACGCAGGAAAGAACATGTGAGCAAAAGGCCAGCAAAAGGCCAGGAACCGTAAA
AAGGCCGCGTTGCTGGCGTTTTTCCATAGGCTCCGCCCCCCTGACGAGCATCACAAAAATCGACGCTCAAG
TCAGAGGTGGCGAAACCCGACAGGACTATAAAGATACCAGGCGTTTCCCCCTGGAAGCTCCCTCGTGCGCT
CTCCTGTTCCGACCCTGCCGCTTACCGGATACCTGTCCGCCTTTCTCCCTTCGGGAAGCGTGGCGCTTTCT
CATAGCTCACGCTGTAGGTATCTCAGTTCGGTGTAGGTCGTTCGCTCCAAGCTGGGCTGTGTGCACGAACC
CCCCGTTCAGCCCGACCGCTGCGCCTTATCCGGTAACTATCGTCTTGAGTCCAACCCGGTAAGACACGACT
TATCGCCACTGGCAGCAGCCACTGGTAACAGGATTAGCAGAGCGAGGTATGTAGGCGGTGCTACAGAGTTC
TTGAAGTGGTGGCCTAACTACGGCTACACTAGAAGGACAGTATTTGGTATCTGCGCTCTGCTGAAGCCAGT
TACCTTCGGAAAAAGAGTTGGTAGCTCTTGATCCGGCAAACAAACCACCGCTGGTAGCGGTGGTTTTTTTG
TTTGCAAGCAGCAGATTACGCGCAGAAAAAAAGGATCTCAAGAAGATCCTTTGATCTTTTCTACGGGGTCT
GACGCTCAGTGGAACGAAAACTCACGTTAAGGGATTTTGGTCATGAGATTATCAAAAAGGATCTTCACCTA
GATCCTTTTAAATTAAAAATGAAGTTTTAAATCAATCTAAAGTATATATGAGTAAACTTGGTCTGACAGTT
ACCAATGCTTAATCAGTGAGGCACCTATCTCAGCGATCTGTCTATTTCGTTCATCCATAGTTGCCTGACTC
CCCGTCGTGTAGATAACTACGATACGGGAGGGCTTACCATCTGGCCCCAGTGCTGCAATGATACCGCGAGA
CCCACGCTCACCGGCTCCAGATTTATCAGCAATAAACCAGCCAGCCGGAAGGGCCGAGCGCAGAAGTGGTC
CTGCAACTTTATCCGCCTCCATCCAGTCTATTAATTGTTGCCGGGAAGCTAGAGTAAGTAGTTCGCCAGTT
AATAGTTTGCGCAACGTTGTTGCCATTGCTACAGGCATCGTGGTGTCACGCTCGTCGTTTGGTATGGCTTC
ATTCAGCTCCGGTTCCCAACGATCAAGGCGAGTTACATGATCCCCCATGTTGTGCAAAAAAGCGGTTAGCT
CCTTCGGTCCTCCGATCGTTGTCAGAAGTAAGTTGGCCGCAGTGTTATCACTCATGGTTATGGCAGCACTG
CATAATTCTCTTACTGTCATGCCATCCGTAAGATGCTTTTCTGTGACTGGTGAGTACTCAACCAAGTCATT
CTGAGAATAGTGTATGCGGCGACCGAGTTGCTCTTGCCCGGCGTCAATACGGGATAATACCGCGCCACATA
GCAGAACTTTAAAAGTGCTCATCATTGGAAAACGTTCTTCGGGGCGAAAACTCTCAAGGATCTTACCGCTG
TTGAGATCCAGTTCGATGTAACCCACTCGTGCACCCAACTGATCTTCAGCATCTTTTACTTTCACCAGCGT
TTCTGGGTGAGCAAAAACAGGAAGGCAAAATGCCGCAAAAAAGGGAATAAGGGCGACACGGAAATGTTGAA
TACTCATACTCTTCCTTTTTCAATATTATTGAAGCATTTATCAGGGTTATTGTCTCATGAGCGGATACATA
TTTGAATGTATTTAGAAAAATAAACAAATAGGGGTTCCGCGCACATTTCCCCGAAAAGTGCCACCTGACGT
CTAAGAAACCATTATTATCATGACATTAACCTATAAAAATAGGCGTATCACGAGGCCCTTTCGTCTTCAC
